# Supplementary material for: On the surface or down below: Field observations reveal a high degree of surface activity in a burrowing crayfish, the Little Brown Mudbug (Lacunicambarus thomai)
Source: PLoS One. 2022 Oct 14;17(10):e0273540. doi: 10.1371/journal.pone.0273540 (PMC9565396; doi:10.1371/journal.pone.0273540)
Supplement: S1 Table — (DOCX) [file pone.0273540.s004.docx]

**S1 Table. The most likely model predicting the activity of burrowing crayfish based on time, humidity, temperature, an interaction between time and humidity, and an interaction between humidity and temperature.** For each model, degrees of freedom (d.f.), the corrected Akaike information criterion (AICc), Akaike weight (*w*), and the log likelihood (ll) are reported. Models were ranked according to their corrected Akaike information criterion (AICc). Besides the null model, only models with a *w* > 0.00 are reported.

| model | d.f. | ll | AICc | DAICc | *w* |
| --- | --- | --- | --- | --- | --- |
| Time + humidity + temperature + time*humidity + humidity*temperature | 8 | -367.90 | 752.00 | 0.00 | 0.42 |
| Time + humidity + temperature + humidity*temperature | 7 | -369.90 | 753.96 | 1.96 | 0.16 |
| Time + humidity + temperature + time*humidity + time*temperature + humidity*temperature | 9 | -367.88 | 754.01 | 2.00 | 0.15 |
| Time + humidity + temperature + time*humidity + time*temperature + humidity*temperature + time*humidity*temperature | 10 | -367.08 | 754.47 | 2.47 | 0.12 |
| Humidity + temperature + humidity*temperature | 6 | -371.61 | 755.34 | 3.34 | 0.08 |
| Time + humidity + temperature + time*temperature + humidity*temperature | 8 | -369.70 | 755.61 | 3.61 | 0.07 |
| null | 3 | -482.07 | 970.17 | 218.16 | 0.00 |
