# Supplementary material for: On the surface or down below: Field observations reveal a high degree of surface activity in a burrowing crayfish, the Little Brown Mudbug (Lacunicambarus thomai)
Source: PLoS One. 2022 Oct 14;17(10):e0273540. doi: 10.1371/journal.pone.0273540 (PMC9565396; doi:10.1371/journal.pone.0273540)
Supplement: S2 Table — (DOCX) [file pone.0273540.s005.docx]

**S2 Table. Coefficients and standard errors (SE) for the model predicting the activity of *L. thomai* based on full model averaging.** Coefficients are reported in order based on their absolute values. Humidity was the strongest predictor of activity. Thus, the activity of *L. thomai* was negatively related to the degree of environmental humidity.

| Independent variable | Coefficient | SE |
| --- | --- | --- |
| Humidity | -2.908 | 1.36 |
| Humidity*temperature | 0.057 | 0.019 |
| Temperature | -0.045 | 0.012 |
| Time*humidity | 0.005 | 0.089 |
| Time | 0.003 | 0.053 |
| Time*humidity*temperature | < -0.001 | 0.001 |
| Time*temperature | < 0.001 | 0.001 |
