# Supplementary material for: On the surface or down below: Field observations reveal a high degree of surface activity in a burrowing crayfish, the Little Brown Mudbug (Lacunicambarus thomai)
Source: PLoS One. 2022 Oct 14;17(10):e0273540. doi: 10.1371/journal.pone.0273540 (PMC9565396; doi:10.1371/journal.pone.0273540)
Supplement: S3 Table — (DOCX) [file pone.0273540.s006.docx]

**S4 Table. Results from Chi-squared test for the number of behaviors that occur during the day and versus the night.** For each behavior, we report the degrees of freedom (d.f.), Chi-squared value (χ2), and *p* value. All behaviors showed significant differences between the number of times the behavior was observed during the day or night.

| Behavior |  | | |
| --- | --- | --- | --- |
|  | d.f. | χ2 | *p* |
| Relaxed | 1 | 178.94 | 0.002 |
| Guard | 1 | 15.01 | < 0.001 |
| Forage | 1 | 171.07 | < 0.001 |
| Excavate | 1 | 92.45 | < 0.001 |
| Hunt | 1 | 9.32 | < 0.001 |
| Under | 1 | 66.16 | < 0.001 |
| All behaviors | 5 | 269.29 | < 0.001 |
