# Supplementary figures and images for: On the surface or down below: Field observations reveal a high degree of surface activity in a burrowing crayfish, the Little Brown Mudbug (Lacunicambarus thomai)
Source: PLoS One. 2022 Oct 14;17(10):e0273540. doi: 10.1371/journal.pone.0273540 (PMC9565396; doi:10.1371/journal.pone.0273540)

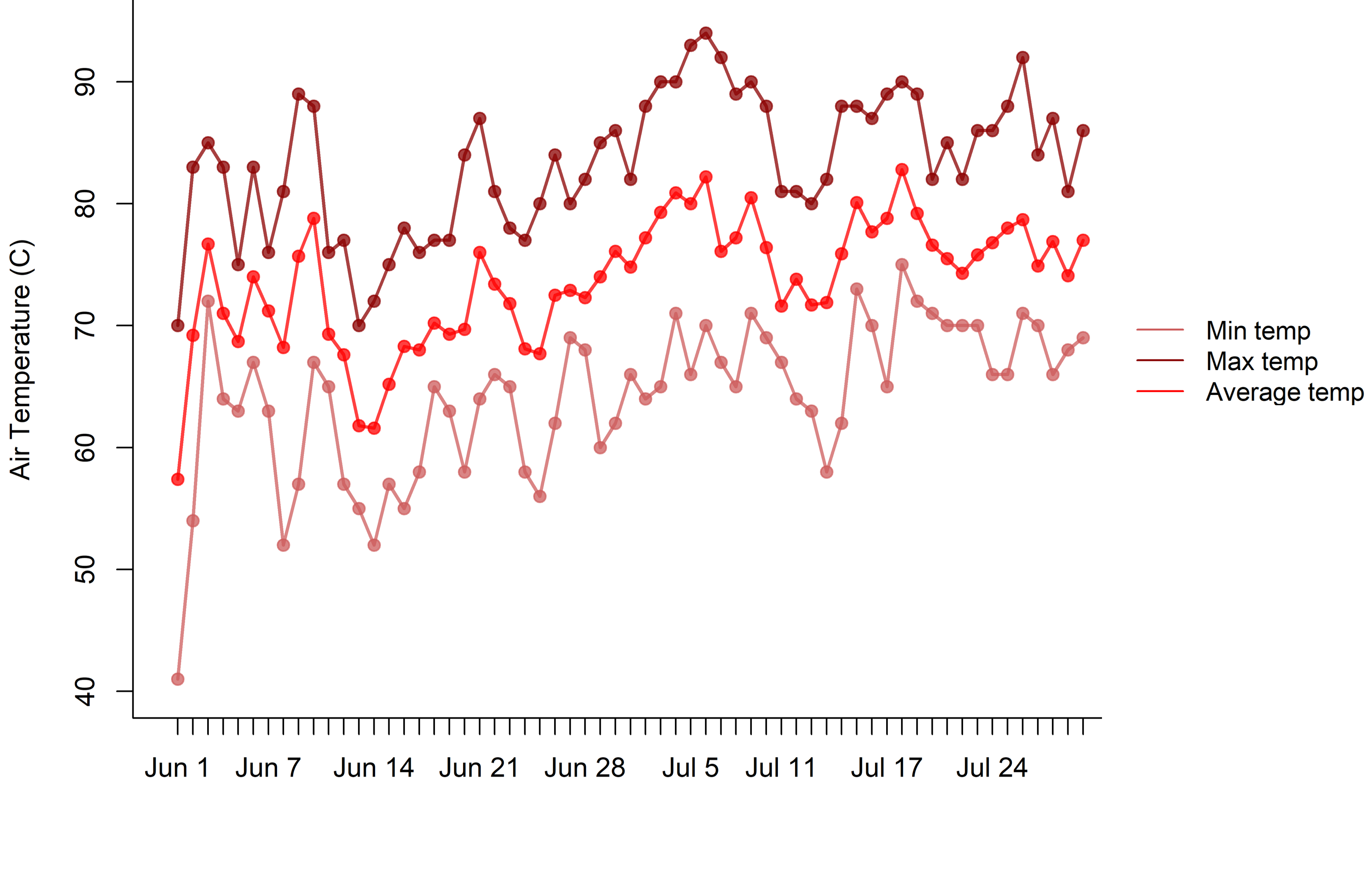

Supplement: S1 Fig — (PNG) [file pone.0273540.s008.png]

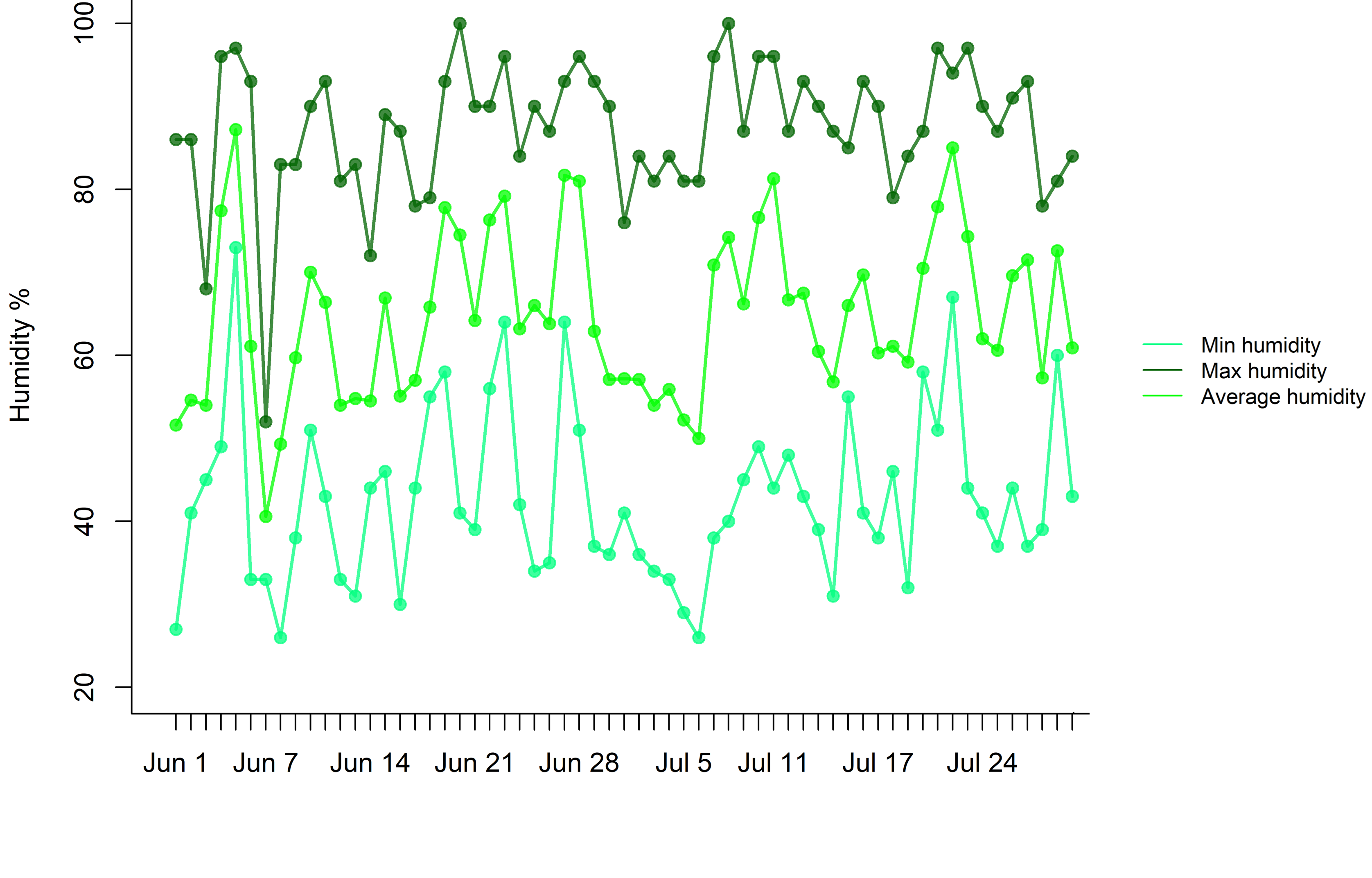

Supplement: S2 Fig — (PNG) [file pone.0273540.s009.png]

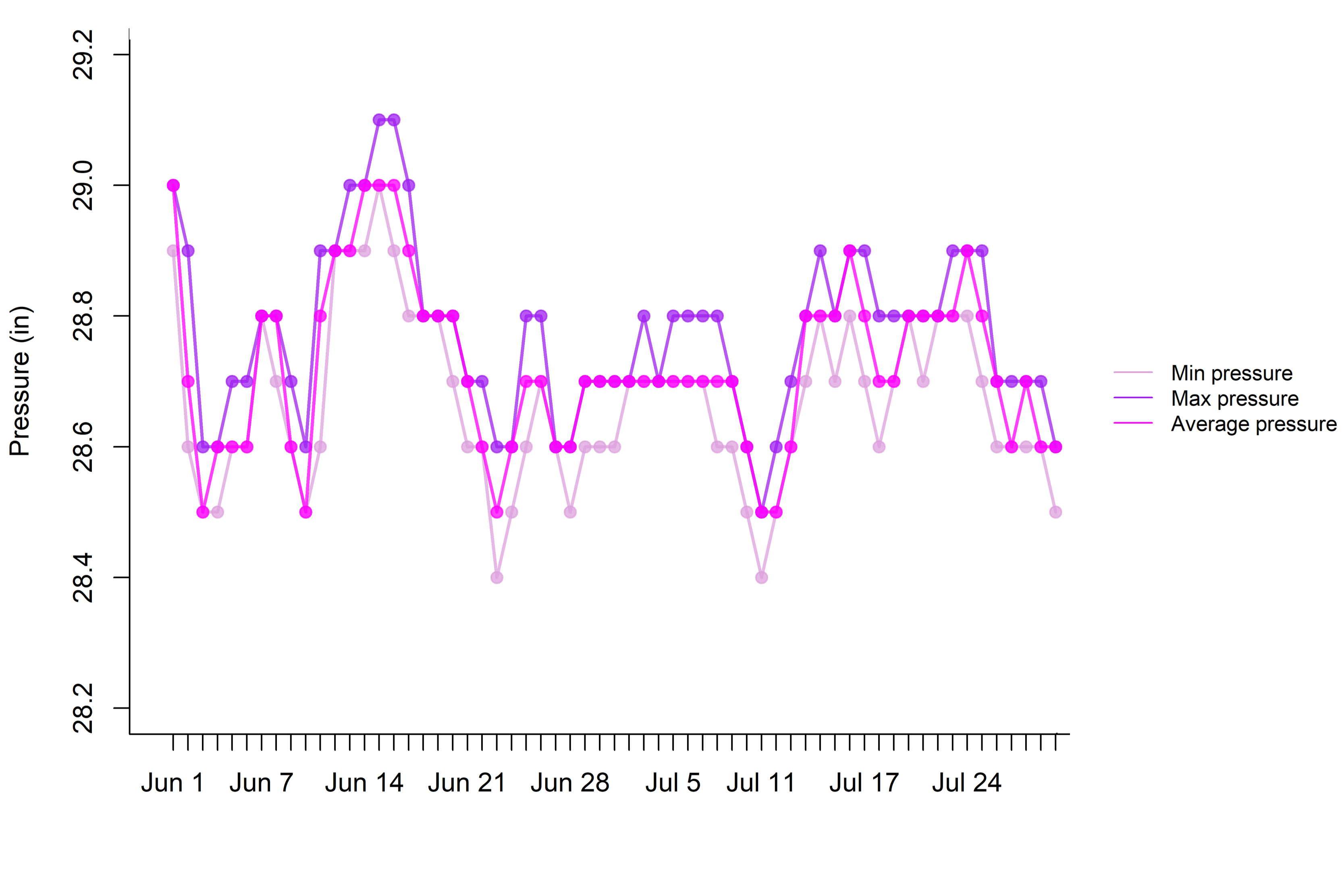

Supplement: S3 Fig — (PNG) [file pone.0273540.s010.png]

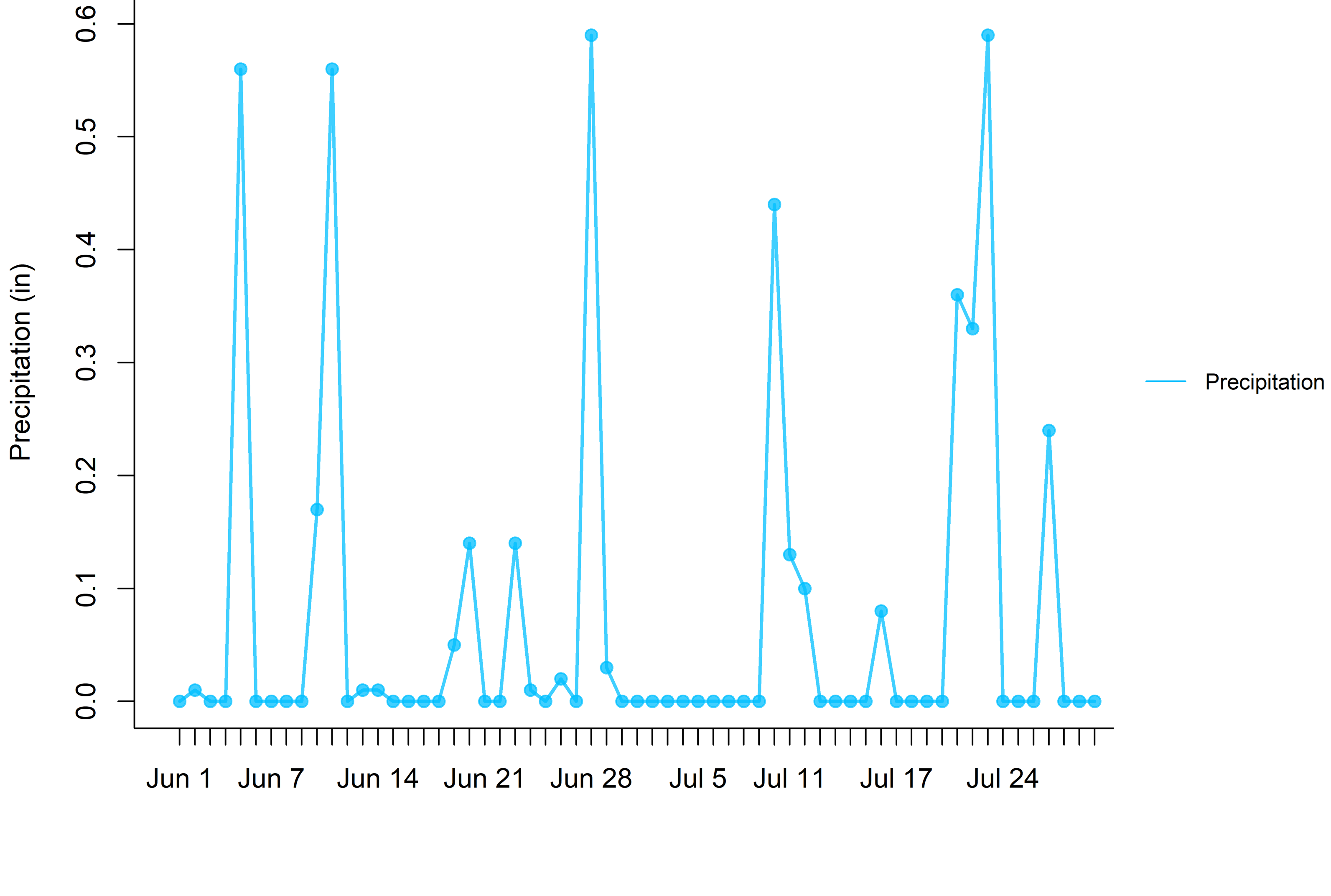

Supplement: S4 Fig — (PNG) [file pone.0273540.s011.png]
