# Supplementary material for: On the surface or down below: Field observations reveal a high degree of surface activity in a burrowing crayfish, the Little Brown Mudbug (Lacunicambarus thomai)
Source: PLoS One. 2022 Oct 14;17(10):e0273540. doi: 10.1371/journal.pone.0273540 (PMC9565396; doi:10.1371/journal.pone.0273540)
Supplement: Attachment — Submitted filename: PONE-D-21-39709_R1_LFB.pdf [file pone.0273540.s013.pdf]

PLOS ONE

On the surface or down below: Field observations reveal a high degree of surface activity in a burrowing crayfish, the Little Brown Mudbug (Lacunicambarus thomai)

--Manuscript Draft--

**Manuscript Number:**	PONE-D-21-39709R1
**Article Type:**	Research Article
**Full Title:**	On the surface or down below: Field observations reveal a high degree of surface activity in a burrowing crayfish, the Little Brown Mudbug (Lacunicambarus thomai)
**Short Title:**	Burrowing Crayfish Behavior
**Corresponding Author:**	Kaine M Diehl<br>West Liberty University<br>West liberty, West Virginia UNITED STATES
**Keywords:**	primary burrowing crayfish; Behavioral Ecology; Behavior; crustacean; ethogram
**Abstract:**	Opposed to most crayfish species that inhabit permanent bodies of water, a unique burrowing lifestyle has evolved several times throughout the crayfish phylogeny. Burrowing crayfish are considered to be semi-terrestrial, as they burrow to the groundwater—creating complex burrows that occasionally reach 3 m in depth. Because burrowing crayfishes spend most of their lives within their burrow, we lack a basic understanding of the behavior and natural history of these species. However, recent work suggests that burrowing crayfishes may exhibit a higher level of surface activity than previously thought. In the current study, we conducted a behavioral study of the Little Brown Mudbug, Lacunicambarus thomai using video surveillance to determine their degree of surface activity and behavioral patterns. Throughout 664 hrs of footage, we observed a surprisingly high amount of activity at the surface of their burrows—both during the day and night. The percentage of time that individual crayfish was observed at the surface ranged from 20.55% to 68.75% per individual, with an average of 42.48% of the time spent at the surface across all crayfish. Additionally, we created an ethogram based on six observed behaviors and found that each behavior had a strong circadian effect. For example, we only observed a single observation of foraging on vegetation during the day, whereas 270 observations of this behavior were documented at night. Overall, our results suggest that burrowing crayfishes may exhibit higher levels of surface activity than previously thought. To increase our understanding of burrowing crayfish behaviors ecology, we encourage the continued use of video-recorded observations in the field and the laboratory.
**Order of Authors:**	Kaine M Diehl
	Nicoleena M. Storer
	Hogan D. Wells
	Destinee A. Davis
	Zachary J. Loughman
	Zackary A. Graham
**Opposed Reviewers:**	Jim Stoeckel<br>Associate Professor, Auburn University<br>jimstoeckel@auburn.edu<br>Expertise in burrowing crayfish behavior and ecology
	Zanethia Barnett<br>Research Fisheries Biologist, USDA Forest Service Southern Research Station<br>zanethia.c.barnett@usda.gov<br>Expertise in crayfish ecology
	Mael Glon<br>Lecturer, The Ohio State University<br>glon.1@osu.edu<br>Expertise in burrowing crayfish behavior and taxonomy

Powered by Editorial Manager® and ProduXion Manager® from Aries Systems Corporation

Response to Reviewers:	Dear Dr. Junhu Dai,<br><br>Thank you for allowing us the opportunity to revise our manuscript. The comments from the two reviewers were extremely helpful and made the manuscript much stronger. Based on their suggestions, we incorporated most suggestions to our manuscript.<br><br>Below, you will find a letter detailing how we dealt with each comment of each reviewer. Where applicable, we state what we have changed and the location in which you will find our modifications in our updated manuscript.<br><br>We address each reviewer comment (C#) in individual answers (A#) to keep everything organized.<br><br>Reviewer #1:<br>C1: The authors made an interesting work, continuously filming the surface behavior of crayfish which were thought before to stay mostly or totally in underground burrows. They registered activity both during the day and night along with daily environmental factor variations and analyzed their behavior through the video recordings. While most behavioral studies in crayfish have been done in laboratory settings, this one contributes by rescuing ecological context and opening new perspectives of research. It is mostly well done but there are three general issues to be considered:<br><br>A1: Thank you for the generous comments. We appreciate your suggestions and we have attempted to address all your comments below.<br>____<br>C2: One of the big contributions of this work is to enable discrimination of individual behavior, which is not a simple task in natural observations. Individual data presentations are fine but I suspect that when average analysis are presented, some or most of them are not considering that one individual (#2) with more than double the number of data collection is biasing the results. This can be deduced from some graphs while others do not let us know because description of average calculations are missing in Materials and Methods.<br><br>A2: We agree that analyzing behavioral data presents several issues and that this issue can be exacerbated by having an unequal sample size (263 hrs of footage for crayfish 2 vs 40 hours for crayfish 1). This is why we have chosen to present the majority of our results in terms of the percentages and not raw values. Reporting this information in terms of the raw values would certainly bias our results, as you suggest. However, by reporting this information (Figures 5, 6, and 7) in percentages, we believe that this is the most appropriate way to report our results and adjust for the bias. Furthermore, our manuscript reports relatively few true statistical analyses (and instead opts for more descriptive statistics of what we observed). This aligns with the primary goals of our manuscript—to describe the behavioral diversity of this elusive crayfishes and demonstrate how this new methodology can be lucrative in the field of crustacean behavioral ecology.<br>____<br><br>C3: While behavioral analysis can be made when these crayfish are on surface, nothing can be stated of their underground times. In this sense, it is an error to assign “inactivity” for underground times.<br><br>A3: We agree. Our manuscript can only report of the surface activity, and not any potential underground behaviors. We have altered our language throughout the entire manuscript to account for this comment. We have changed any discussion of “inactivity” to “inactivity at the surface”.<br>____<br><br>C4: Discussion needs to be improved.<br><br>A4: We have taken several of your comments regarding topics that require additional discussion and expanded on them.

Powered by Editorial Manager® and ProduXion Manager® from Aries Systems Corporation

_

C5: Study Site: More basic description is required of the study area around the filming spot, to better explore the results. It is said that most species (tertiary and secondary burrowing crayfish) inhabit lotic and lentic environments but it is not clear how far are the Primary burrowing species of this study from water bodies, for instance. It is said in the Introduction that “Lacunicambarus thomai is a burrowing crayfish species with a high propensity to inhabit burrows in marshes, roadside ditches, and flooded fields (38). Populations of L. thomai often live in localized colonies with conspecifics and inhabit burrows that are relatively simple but can nonetheless be up to 1-1.5 m deep (38,39)”. However, nothing, no information is provided of the specific area where the study took place: proximity from rivers, vegetation types and cover densities, average ranges of daily environmental factor variations, Latitudinal coordinates.

A5: We agree that information on our study site was lacking in the original version of the manuscript. We have now included a detailed description of the location in which we conducted this study (see Lines 127-134). Because our sampling location is on a residential property, we choose to not report exact coordinates.

_

C6: Burrows were selected and filmed. How far is one burrow from the other? Inform the average land area inside which the 6 burrows were located. One individual is associated to each burrow – is that an assumption or are there evidences that they are solitary? Furthermore, it is said that burrows may have more than one entrance (Line 67), how was this issue treated here?

A6: We have updated this information within our manuscript. We now provide information regarding how far away one burrow was from one another (Lines 131-132), as well as our assumption that each burrow was occupied by a single crayfish (Lines 141-144). We also clarify how we dealt with the issue with burrows with more than a single entrance (Lines 151-153).

_

C7: It is said in Statistical Analysis that none of the independent variables (humidity, temperature, and time) were strongly correlated (all $r < 0.6$). A representative graph depicting an average 24h variation of environmental factors in the studied season would be informative in Supplemental Material. How was time included in the models, as a categorical or continuous variable? A continuous variable with linear increase (such as a sequence from 0 to 23) would create artificial associations in the model. Please evaluate, based on the statistical parameters found in Table 3, if it is not enough to use a simple model without interaction between time, humidity and temperature to understand the influence of environmental factors on the surface activity. The reason for this question is that the complexity of the best-fit model seems to have inhibited any discussion about the analysis in the end.

A7: We have now included 4 new figures in the supplemental materials (Figures S1, S2, S3, and S4) which display the relevant environmental data during our study period.

In the original manuscript, we reported that there were no strong statistical correlations between humidity, temperature, and time (as you mentioned). In our environmental analysis, time was coded as a continuous variable. We tested for the correlations beforehand to potentially deal with any artificial associations that you hint at. However, because of the low correlation values, we decided to proceed with using time as a continuous variable. Furthermore, the fact that time is not a strong predictor of activity (Table 4) suggests that there is no underlying association between these variables.

Our best fit model was quite complex, as you note. It was a model with 3 single parameters, and two interaction parameters. This why is we not only conducted a model-selection procedure (results in Table 3), but also the multi-model average procedure (results in Table 4). The results from our full model averaging technique allow us to evaluate all of the models together and construct effect sizes and weights

Powered by Editorial Manager® and ProduXion Manager® from Aries Systems Corporation

	for each of the terms in our model. This is why we come to the conclusion that humidity had the strongest overall effect (Table 4) compared to the other models. We have now included a better discussion of this result in our discussion section (see A14).<br>———<br>C8: The Results section start with “Hourly Activity” (which should be “Hourly surface activity”) and Figure 4, but there is no explanation as to how this was calculated. It is explained in Statistical Analysis that for an individual to be considered active on surface in one specific hour and day, it needs to be seen in any time point within that hour in that day. Then, how was the group average/percentage calculated taking into account that each contributing individual was registered for a different number of days? Individuals that had more filmed days should not weight more. Finally, Legend Figure 4 needs to inform that averages were calculated taking into account all individuals and all days.<br><br>A8: We have changed this section to “Hourly Surface Activity” as you suggest.<br><br>We have clarified how this was calculated in the methods sections (Lines 201-209). Again, we report these values in terms of percentages, and not the raw number of hours each crayfish was active to avoid any potential sampling biases as you suggest.<br><br>We have also updated the legend for Figure 4 to include those averages were calculated by taking into account for all individuals and all days throughout our study.<br>———<br>C9: An explicit description of calculation should also be added to “percentage of time spent active throughout daytime” and “ proportion of time spent on surface”. In Table 5, how was the “mean duration” of each behavior calculated, taking into account individuals and number of days each individual was filmed?<br><br>A9: We have updated our methods to also include information as to how we calculated these percentages. By reporting these values in percentages, we avoid any issues based on the number of hours each crayfish was sampled and filmed.<br><br>We have updated the legend in figure 5 to describe that our mean durations were calculated based on all crayfish. This data is reported to be entirely descriptive in nature, and therefore we believe that reporting such means are the most informative way to describe these different behaviors.<br>———<br>C10: In Figures 5, 6 and 7, it is interesting to show the “combined” proportions. However, it is biased by individual 2, which was filmed for more days. Could an unbiased calculation be made here?<br><br>A10: Reporting our results in terms of percentages is the best way to provide an unbiased calculation for these figures, which are primarily meant to be descriptive and exploratory in nature. If we would have reported the data from the raw amount of time that each behavior/activity was performed, then this would certainly be biased. This is why we chose to reported the data in terms of percentages throughout the manuscript.<br>———<br>C11: In Figure 8 the number of observations is again biased to individual 2.<br><br>A11: Yes, Figure 8 (but not 5, 6, and 7) is biased based on the increased number of observations from individual 2. This figure (and the analysis in general) are describing trends in whether or not a specific behavior was more or less likely to occur at day versus night. We have included information regarding this issue in our manuscript (Lines 201-203). Anecdotally, if you look at the percentages of all behaviors reported in Fig 5, 6, and 7, there is a similar degree of surface activity/behaviors being exhibited by each crayfish, which implies that this bias may be minimally impacting our results.

Powered by Editorial Manager® and ProduXion Manager® from Aries Systems Corporation

_

C12: Figure 5 and associated text: comparison should be between “surface” and “underground”, not between “active” and inactive” because nothing is known about what the crayfish are doing underground. The same for Figure 6: “nighttime on surface” and “daytime on surface”. In Line 281, replace “ active” by “on surface” in “The percentage of time that crayfish were active during the daytime”. In Line 283, “Regarding nighttime activity on surface”. Legend Figure 6: “percentage of time that each individual crayfish spent on surface during the day and the night”.

A12: You are correct; we do not want to mislead the readers. Now, all of our figures have been accounted for the fact that we are only reporting surface activity and that we cannot comment on underground. Activity. Figures 5 and 6 are now updated accordingly. We have also changed the legend for Figure 6

_

C13: Discussion: In contrast to Bearden et al. (2021), this study brings more information about the behavioral complexity of this particular crayfish species. However, the lengthy discussion is mainly descriptive of results. The authors should explore, for instance, what was found in statistical analysis and how could this be connected to the specificity of the studied environment, to take full advantage of the in situ study. Another suggestion is to take Bearden et al. (2021) as a reference, discuss how the results are constrained by the particular season and microhabitat that was covered in this study.

A13: We agree that we could have expanded on our discussion section. Based on many of your suggestions below we believe that the discussion has significantly improved.

_

C14: Humidity was indicated as the most important factor modulating surface appearance in crayfish. This variable, as well as all others were collected from a meteorological station. Any thoughts about the validity of using only macro-environmental measurements in association with behaviors that are restricted to the spatial scale of the entrance of a burrow?

A14: We agree that the macro environmental variables that we relate to surface activity are far from ideal for such studies. We have updated our discussion to point this out and suggest that future studies take into account the variables potential micro-environmental influences within and around the burrow entrances (Lines 406-422).

_

C15: Temperature was shown to be a strong predictor of surface activity. A strong suggestion for future studies is to also consider underground temperature in this analysis. For instance, it has been shown in endothermic subterranean rodents that a combination of external and underground temperatures predict better the episodes of surface emergences (Jannetti et al., Conservation Physiology 2019 v. 7, p. coz044). It is reasonable to assume that similar influence is potentially valid for these crayfish.

A15: This is a great suggestion to add to our discussion section. We have now included that looking at the underground temperature variation is a fruitful area for future directions (Lines 416-422).

_

C16: This crayfish display clear postural signatures that enable behavioral identification through the relative position of body coordinates (Figure 3). This could potentially be used in future automated video analysis of activity patterns.

A16: That is a great suggestion to add the discussion. We have included these

postural changes to be used in the identification of automated video analysis in the future (Lines 511-513).

_

C17: Fig.2C: view of the camera

A17: We have edited this sentence accordingly.

_

C18: Line 248: remove extra “were”

A18: We have removed the extra “were”

_

C19: Line 319: “every single behavior was more likely to occur during day or night compared to another” An alternative could be “daily phase”

A19: We believe that the current wording provides for clarity because “daily phase” may be confused with “day”.

_

C20: Table 4: what does this mean? “the activity of L. thomai was negatively related to the activity of
crayfish.”

A20: This was a typo and should have read “Thus, the activity of L. thomai was negatively related to the degree of environmental humidity.” We have made this change to the Table 4 text. Thank you.

_

Reviewer #2:
C21: I understand that behaviour of strictly burrowing crayfish is difficult to study, so the information presented by the authors is surely interesting, and novel. However, the entire manuscript is based on only six individuals that were not even characterized. So, data are very preliminary and should be interpreted more cautiously. Behavioural categories should be also analysed together, considering that are dependent data. I am sorry not to be more positive, but a more specialised journal seems to be more appropriate for the manuscript.

A21: We agree that the fact that our study being only conducted on six individuals is a limitation to our study. Low sample sizes are typical for this type of work, because of the time intensive nature and complexities of naturalistic studies (see 6 individuals reported in Janettii et al. 2019) Although this work was only conducted on six individuals (that have unknown demographic information), we believe that the amount and nature of our data is worthy of publication.

In the previous and updated versions of our manuscript, we attempt not to overstate our results as they are preliminary and exploratory in nature. Furthermore, although we do not report the demographic data from these crayfishes (sex, etc), there is no data that has alluded to sex differences in burrowing crayfish behavior. We do agree that this is an interesting and important angle for future studies though, so we have included that this information should be investigated in the future (Lines 507-508).

Despite these limitations, we still believe that we provide a novel methodology and interesting results (which reviewer 1 highlights) that will be of interest to a wide audience. Because the scope of PLOS ONE is to publish papers based on their scientific validity and methodology, we believe that our study is of interest to an audience outside of smaller, taxonomic focused journals.

We respond to the comment on behavioral categories being analyzed together below (see A27).

_

C22: Lines 50-52, 109: for the readers it would be interesting to specify that these species are North American

A22: We have edited Lines 50-52 to clarify that these are North American species (Line 50-51)

_

C23: Line 67: maybe opening is more suitable than portal

A23: Portal is a term burrowed from the literature on mammalian burrowing behavior and is widely used to refer to crayfish burrow openings. Therefore, we prefer to keep this language consistent with prior published papers. We have included a few examples below.

Glon, M. G., Adams, S. B., Loughman, Z. J., Myers, G. A., Taylor, C. A., & Schuster, G. A. (2020). Two new species of burrowing crayfish in the genus Lacunicambarus (Decapoda: Cambaridae) from Alabama and Mississippi. Zootaxa, 4802(3), 401-439.

Loughman, Z. J. (2010). Ecology of Cambarus dubius (upland burrowing crayfish) in north-central West Virginia. Southeastern Naturalist, 9(sp3), 217-230.

_

C24: Lined 128-130: so how many burrows were checked before selecting only six? How about the density of the burrows in the study area?

A24: We have now included much more information regarding our study location. Based on the relatively small number of burrows at the study location, we choose to only focus on a small sample size but to collect as much data as possible on each of these adult individuals.

_

C25: Line 132: crayfish could have been attracted out of the burrows with some baits after the footage to characterize them (for hunt behaviour it is reported they leave the burrow for example)

A25: Yes, that is true. Unfortunately, we did not capture these individuals. We have included information how future studies need to explore how different demographics may exhibit such behaviors differently (lines 50-51).

_

C26: Line 182: were the behavioural data checked for normality?

A26: Yes, all data and model fits were checked for normality. We have now included this information in the manuscript (Lines 197-199).

_

C27: Lines 218-222: those behaviours are dependent each other, so it is better to analyse them together because when crayfish are guarding, for example, they are not feeding or hunting

A27: This comment highlights the complexities of working with behavioral data, because an organism cannot perform more than a single behavior at once. Therefore, this is why we chose to primarily focus on broad, descriptive statistics throughout our study. Because of the novelty of these findings and the potential impact on our

understanding of crustacean behavioral ecology, we believe that this is the proper way to analyze report our data at this stage.

_

C28: Line 235: please delete during the study

A28: We have deleted this phrase.

_

C29: Line 270: please 5 not in italics

A29: We have unitalicized this number.

_

C30: I suggest merging Fig 5 and 6 in one figure

A30: We prefer to keep these figures separate as to avoid confusion between the messages of figure 5 (observed at surface vs. underground) and figure 6 (observed at surface during the day versus observed at surface during the night).

_

C31: Table 5: please report all the duration in seconds. Moreover, please change during with duration in the caption

A31: We have changed the table to report each behavior in. We have changed during to duration as you suggested.

_

C32: Tables should be better drafted and presented

A32: Without any comments on the issues of the tables, we are unsure what to change base.

_

C33: Line 343: please correct five

A33: We change made this change.

_

C34: Line 345: please better rephrase this sentence

A34: We have rephrased this sentence accordingly (Lines 366-368).

_

C35: Line 356: Loughman et al. 2018 is not present in the bibliography; 46 is Loughman et al. 2015

A35: Thank you for noticing this issue. We meant Loughman et al. 2015 and we have made this change.

_

C36: Line 371, 448: please consider that only six individuals were observed, so I suggest being more cautious in this statement

A36: We agree that we need to be more cautious with these statements. We have

	added additional information to these sections based on your comments (see Lines 394-395 and Lines 494-495).<br>____<br>_<br>C37: Line 381: please do not use intricacies but richness or diversity<br><br>A37: We have changed the work intricacies to richness as you have suggested.<br>____<br>_<br>C38: Line 415: please correct “is required”<br><br>A38: We have changed “are required” to “is required”<br>____<br>_<br>C39: Line 436: I think it is “use its claws to push the mud”<br><br>A39: Thank you for catching this error. We have fixed the typo.
**Additional Information:**	
**Question**	**Response**
**Financial Disclosure**<br><br>Enter a financial disclosure statement that describes the sources of funding for the work included in this submission. Review the submission guidelines for detailed requirements. View published research articles from *PLOS ONE* for specific examples.<br><br>This statement is required for submission and **will appear in the published article** if the submission is accepted. Please make sure it is accurate.	The author(s) received no specific funding for this work

**Unfunded studies**<br>Enter: *The author(s) received no specific funding for this work.*<br><br>**Funded studies**<br>Enter a statement with the following details:<br>• Initials of the authors who received each award<br>• Grant numbers awarded to each author<br>• The full name of each funder<br>• URL of each funder website<br>• Did the sponsors or funders play any role in the study design, data collection and analysis, decision to publish, or preparation of the manuscript?<br>• **NO** - Include this sentence at the end of your statement: *The funders had no role in study design, data collection and analysis, decision to publish, or preparation of the manuscript.*<br>• **YES** - Specify the role(s) played.<br><br>* typeset	
**Competing Interests**<br><br>Use the instructions below to enter a competing interest statement for this submission. On behalf of all authors, disclose any competing interests that could be perceived to bias this work—acknowledging all financial support and any other relevant financial or non-financial competing interests.<br><br>This statement is required for submission and **will appear in the published article** if the submission is accepted. Please make sure it is accurate and that any funding sources listed in your Funding Information later in the submission form are also declared in your Financial Disclosure statement.<br><br>View published research articles from *PLOS ONE* for specific examples.	The authors have declared that no competing interests exist

Powered by Editorial Manager® and ProduXion Manager® from Aries Systems Corporation

**NO authors have competing interests**<br><br>Enter: *The authors have declared that no competing interests exist.*<br><br>**Authors with competing interests**<br><br>Enter competing interest details beginning with this statement:<br><br>*I have read the journal's policy and the authors of this manuscript have the following competing interests: [insert competing interests here]*<br><br>* typeset	
**Ethics Statement**<br><br>Enter an ethics statement for this submission. This statement is required if the study involved:<br><br>• Human participants<br>• Human specimens or tissue<br>• Vertebrate animals or cephalopods<br>• Vertebrate embryos or tissues<br>• Field research<br><br>Write "N/A" if the submission does not require an ethics statement.<br><br>General guidance is provided below. Consult the submission guidelines for detailed instructions. **Make sure that all information entered here is included in the Methods section of the manuscript.**	N/A

**Format for specific study types**<br><br>**Human Subject Research (involving human participants and/or tissue)**<br>• Give the name of the institutional review board or ethics committee that approved the study<br>• Include the approval number and/or a statement indicating approval of this research<br>• Indicate the form of consent obtained (written/oral) or the reason that consent was not obtained (e.g. the data were analyzed anonymously)<br><br>**Animal Research (involving vertebrate animals, embryos or tissues)**<br>• Provide the name of the Institutional Animal Care and Use Committee (IACUC) or other relevant ethics board that reviewed the study protocol, and indicate whether they approved this research or granted a formal waiver of ethical approval<br>• Include an approval number if one was obtained<br>• If the study involved *non-human primates*, add *additional details* about animal welfare and steps taken to ameliorate suffering<br>• If anesthesia, euthanasia, or any kind of animal sacrifice is part of the study, include briefly which substances and/or methods were applied<br><br>**Field Research**<br><br>Include the following details if this study involves the collection of plant, animal, or other materials from a natural setting:<br>• Field permit number<br>• Name of the institution or relevant body that granted permission	
**Data Availability**<br><br>Authors are required to make all data underlying the findings described fully available, without restriction, and from the time of publication. PLOS allows rare exceptions to address legal and ethical concerns. See the PLOS Data Policy and FAQ for detailed information.	Yes - all data are fully available without restriction

Powered by Editorial Manager® and ProduXion Manager® from Aries Systems Corporation

A Data Availability Statement describing where the data can be found is required at submission. Your answers to this question constitute the Data Availability Statement and **will be published in the article**, if accepted.<br><br>**Important:** Stating 'data available on request from the author' is not sufficient. If your data are only available upon request, select 'No' for the first question and explain your exceptional situation in the text box.<br><br>Do the authors confirm that all data underlying the findings described in their manuscript are fully available without restriction?	
**Describe where the data may be found in full sentences. If you are copying our sample text, replace any instances of XXX with the appropriate details.**<br><br>• If the data are **held or will be held in a public repository**, include URLs, accession numbers or DOIs. If this information will only be available after acceptance, indicate this by ticking the box below. For example: *All XXX files are available from the XXX database (accession number(s) XXX, XXX.*).<br>• If the data are all contained **within the manuscript and/or Supporting Information files**, enter the following: *All relevant data are within the manuscript and its Supporting Information files.*<br>• If neither of these applies but you are able to provide **details of access elsewhere**, with or without limitations, please do so. For example:<br><br>*Data cannot be shared publicly because of [XXX]. Data are available from the XXX Institutional Data Access / Ethics Committee (contact via XXX) for researchers who meet the criteria for access to confidential data.*<br><br>*The data underlying the results presented in the study are available from (include the name of the third party*	All data will be uploaded to Dryad Digital Repository

Powered by Editorial Manager® and ProduXion Manager® from Aries Systems Corporation

and contact information or URL).<br>• This text is appropriate if the data are owned by a third party and authors do not have permission to share the data.<br><br>* typeset	
Additional data availability information:	Tick here if the URLs/accession numbers/DOIs will be available only after acceptance of the manuscript for publication so that we can ensure their inclusion before publication.

Powered by Editorial Manager® and ProduXion Manager® from Aries Systems Corporation

WEST LIBERTY UNIVERSITY

December 16, 2021

Dear Editor,

Please consider our research article "On the surface or down below: Field observations reveal a high degree of surface activity in a burrowing crayfish, the Little Brown Mudbug (*Lacunicambarus thomai*)" for publication in the *PLOS ONE*.

Opposed to most crayfish species that inhabit permanent bodies of water, a unique burrowing lifestyle has evolved several times throughout the crayfish phylogeny. Burrowing crayfish are considered to be semi-terrestrial, as they burrow to the groundwater—creating complex burrows that occasionally reach 3 m in depth. Because burrowing crayfishes spend most of their lives within their burrow, we lack a basic understanding of the behavior and natural history of these species. However, recent work suggests that burrowing crayfishes may exhibit a higher level of surface activity than previously thought.

In the current study, we conducted a behavioral study of the Little Brown Mudbug, *Lacunicambarus thomai* using video surveillance to determine their degree of surface activity and behavioral patterns. Our study is the first to implement this technique to study burrowing crayfish behavior. Throughout 664 hrs of footage, we observed a surprisingly high amount of activity at the surface of their burrows—both during the day and night. The percentage of time that individual crayfish were observed at the surface ranged from 20.55% to 68.75% per individual, with an average of 42.48% of the time spent at the surface across all crayfish. Additionally, we created an ethogram based on six observed behaviors and found that each behavior had a strong circadian effect. Overall, our results suggest that burrowing crayfishes may exhibit higher levels of surface activity than previously thought.

Given that our work highlights the behavioral biology of an understudied group of organisms, as well as gives clear directions for future research on this topic, we believe that our manuscript will be of high interest for the readers of the *PLOS ONE*.

Based on their expertise in animal behavior, we suggest Stefano Grignolio, Brock Fenton, or Cristiano Bertolucci as appropriate editors for our manuscript.

Sincerely,

Kaine Diehl

Graduate Researcher

West Liberty University

Manuscript Click here to access/download;Manuscript;Manuscript.docx

On the surface or down below: Field observations reveal a high degree of surface activity in a burrowing crayfish, the Little Brown Mudbug (*Lacunicambarus thomai*)

Kaine M. Diehl [1], Nicoleena M. Storer [1], Hogan D. Wells [1], Destinee A. Davis[1], Zachary J. Loughman[1] & Zackary A. Graham[1]*

[1] Department of Organismal Biology, Ecology, & Zoo Science

West Liberty University, 208 University Dr. West Liberty, WV 26074

*Corresponding Author: Zackary.graham@westliberty.edu

Abstract

Opposed to most crayfish species that inhabit permanent bodies of water, a unique burrowing lifestyle has evolved several times throughout the crayfish phylogeny. Burrowing crayfish are considered to be semi-terrestrial, as they burrow to the groundwater—creating complex burrows that occasionally reach 3 m in depth. Because burrowing crayfishes spend most of their lives within their burrow, we lack a basic understanding of the behavior and natural history of these species. However, recent work suggests that burrowing crayfishes may exhibit a higher level of surface activity than previously thought. In the current study, we conducted a behavioral study of the Little Brown Mudbug, *Lacunicambarus thomai* using video surveillance to determine their degree of surface activity and behavioral patterns. Throughout 664 hrs of footage, we observed a surprisingly high amount of activity at the surface of their burrows—both during the day and night. The percentage of time that individual crayfish was observed at the surface ranged from 20.55% to 68.75% per individual, with an average of 42.48% of the time spent at the surface across all crayfish. Additionally, we created an ethogram based on six observed behaviors and found that each behavior had a strong circadian effect. For example, we only observed a single observation of foraging on vegetation during the day, whereas 270 observations of this behavior were documented at night. Overall, our results suggest that burrowing crayfishes may exhibit higher levels of surface activity than previously thought. To increase our understanding of burrowing crayfish behaviors ecology, we encourage the continued use of video-recorded observations in the field and the laboratory.

Keywords: primary burrowing crayfish, behavioral ecology, behavior, crustacean, ethogram

Introduction

Crayfish are a diverse group of freshwater crustaceans with over 700 species globally (1,2). Many crayfishes are considered to be model organisms for behavioral biology due to their ease of capture, suitability for laboratory studies, simple neural architecture, and complex behavioral repertoires (3–7). However, this work stems from studies of a few model species from North America (i.e., *Procambarus clarkii*, *Procambarus virginalis*, *Faxonius virilis*); all of which inhabit lentic and lotic aquatic environments. Although most crayfish species inhabit surface water systems like streams, lakes, rivers, ponds, and marshes, crayfishes have repeatedly evolved a semi-terrestrial burrowing lifestyle throughout their phylogeny (8–10). Despite all species of crayfish possessing the ability to burrow to some degree (11), the complexity and reliance on these burrows vary greatly both within and between species (12,13).

Crayfishes have historically been assigned to three different ecological classifications based on their burrowing ability and their reliance on their burrows: tertiary burrowing species, secondary burrowing species, and primary burrowing species (12,14). Tertiary burrowing crayfishes are species which rely on permanent bodies of water and are only capable on constructing relatively simple burrows underneath benthic substrates. Secondary burrowing species can also inhabit permanent bodies of water, but during periods of drawdown they are capable of excavating complex burrows to access groundwater. Primary burrowing species (hereafter referred to as burrowing crayfish) are often considered semi-terrestrial as they are highly reliant on their burrows for their entire life; as they potentially serve as a location for breeding, refuge from predators, and as a location to acquire and cache food (12,13). Burrowing crayfish burrows are often extremely complex and may have several entrances (i.e., portals),

tunnels, and chambers that can reach several meters deep (12,13). In addition to providing shelter for the crayfish that construct them, such burrows can serve as necessary habitat for other organisms (15–18) and increase soil bioturbation and habitat complexity (19–21).

Compared to our knowledge of tertiary and secondary crayfishes that occur in lentic and lotic environments, our understanding of burrowing crayfish behavior is scant (13,22). Historically, burrowing crayfish were thought to spend nearly their entire lives below the surface, and thought to occasionally leave their burrows for brief terrestrial excursions to search for food or to find a mate (12). Minimal surface activity and their propensity to retreat to their burrow when startled has led to difficulties in capturing and observing burrowing species (23,24). Resultant of their reclusive lifestyle, our understanding of burrowing crayfish activity patterns and behavior is minimal, with much of the work on these species being derived from *in situ* natural history observations (12,25–28) and not systematic investigations. However, despite these difficulties, semi-naturalistic mesocosms and custom-built observation chambers have been useful in the study of burrowing crayfish behavior (29–33).

Burrowing crayfish are considered to be nocturnal (34), which further complicates our ability to adequately study their behavior. But in some instances, burrowing crayfish are observed at the surface of their burrow during the daytime (12,34–36). Interestingly, recent studies reveal that burrowing crayfish may exhibit a higher level of surface activity than previously thought (35,37). Bearden et al. (2020) conducted the most comprehensive study to date on the activity of burrowing crayfish (37). Using motion-trigger laser photography in two species of burrowing crayfish (*Lacunicambarus erythrodactylus* and *Procambarus holifieldi*), Bearden and colleagues measured activity throughout a one-year period. In their study, activity was considered based on whether the crayfish was visible at the entrance of their burrow (37).

This study confirmed that nocturnal activity occurred most frequently, specifically during times of cool groundwater temperature and warm air temperatures (37). Furthermore, despite observing substantial activity at night for both species, there were many occurrences of daytime surface activity throughout their study.

Bearden *et al*. (2020) provides robust evidence regarding the environmental correlates of activity in these two species and serves as a solid foundation to build off for future studies on burrowing crayfish surface activity. However, the use of photography and not videography limited their insights into crayfish surface behaviors. As such, the photographs made defining behaviors difficult, and limited behavior determination to chimney construction and surface activity (37). As stated previously, Bearden *et al*. (2020) goals were not specific to defining burrowing crayfish surface behaviors, but rather determining if surface activity was correlated to specific environmental correlates. Therefore, the behavioral complexity of burrowing crayfishes still remains unknown. Given their utilization of complex burrow systems and evidence of social behavior (13), burrowing crayfishes species may exhibit complex ritualistic behaviors like non-burrowing crayfishes (3,13,38). However, no study to date has conducted long-term behavioral observations of a burrowing species in a completely natural environment.

Here, we conducted a behavioral study of a burrowing crayfish species through video-recorded field observations. Specifically, we monitored the surface of burrows inhabited by the Little Brown Mudbug, *Lacunicambarus thomai* (Fig 1). *Lacunicambarus thomai* is as a burrowing crayfish species with a high propensity to inhabit burrows in marshes, roadside ditches, and flooded fields (39). Populations of *L. thomai* often live in localized colonies with conspecifics and inhabit burrows that are relatively simple but can nonetheless be up to 1-1.5 m deep (39,40). Compared to most burrowing crayfish species, *L. thomai* and other members of the

genus *Lacunicambarus* seem to exhibit a high degree of surface activity (35,36,39–41), which makes them ideal for our investigations. Overall, we hope to shed light on the surface behavior of *L. thomai* by 1) determining the prevalence of surface activity and 2) creating an ethogram specific to burrowing crayfish, and 3) analyzing the behavioral repertoire and prevalence of behaviors exhibited by *L. thomai*.

Fig 1. Photographs of adult burrowing crayfish, The Little Brown Mudbug, *Lacunicambarus thomai*, exhibiting daylight activity outside of its burrow. The burrow's entrance portal is out of sight due to the extensive leaf litter coverage. Photographs courtesy of John Freudenstein.

Methods

Study site

In June and July 2020, we conducted non-stop 24-hour video surveys within a population of the Little Brown Mudbug, *Lacunicambarus thomai* (Fig 1) in Hickory, Pennsylvania, U.S.A. This population of *L. thomai* is located on the edge of a man-made pond on a private, residential yard. More specifically, our study site was located in a dense patch of grasses on the edge of the pond with no canopy cover. Within this location, the water table remains shallow throughout the year and there is an estimated population of 20 *L. thomai* within an area of ~20 m^2. Burrows were sporadically located throughout the population and were ~1 m to ~ 5 m from one another. No other crayfish species have been collected or identified from this location and no crayfish have ever been collected from the pond. We chose which burrows to record based on evidence of

recent surface activity (activity observed at burrow portal during preliminary night-time observation), as well as whether the burrow portal was visible from an overhead view. Because capturing burrowing crayfish often destroys the burrow and the population was relatively small, we chose to not capture the individuals. Therefore, we do not report morphological or demographic data from the recorded footage (i.e., sex, body size). However, based on the circumference of the burrow tunnels as well as the video footage, all burrows in which we recorded (see below) were from adult *L. thomai*. Importantly, although other burrowing crayfish species are known to exhibit social behavior and have multiple individuals within a single burrow, *Lacunicambarus* spp. seldomly exhibit social behavior and therefore it is assumed that each burrow only housed a single crayfish (41, but see 42).

Because our work was conducted on privately owned property on an unlisted crayfish species and we did not collect any specimens for this project, no specific permits were necessary for our research.

Videography

Overtop each burrow that we recorded, a custom-built video camera mount was constructed by placing a Reolink 5MP video camera inside of a 5-gallon bucket that we mounted with wire approximately 0.5 m above the burrow (Fig 2). These camera mounts protect the camera from the external environment and reduce glare. We recorded each crayfish until their burrow was either capped or until there was a video camera malfunction. If a crayfish burrow contained more than a one portal, we only mounted the video camera overtop the larger, primary portal which is where burrowing crayfish exhibit nighttime activity (*Z. Loughman*, personal communication). In

total, we recorded and reviewed (see below) 664.7 hr of footage from six adult *L. thomai* (Table 1). The length of time recorded for each crayfish ranged from 44.5 6 hr to 263.82 hr (Table 1).

Fig 2. Images representing our custom video surveillance mount. (A) Diagram of the setup of our video surveillance setup used to monitor crayfish behavior. (B) Photograph of the setup *in situ* **C)** Photograph of the view of the camera mounted from inside of the bucket.

Table 1. Dates and total time that each crayfish was filmed throughout the duration of our study. Only a single crayfish (Crayfish 5) was filmed discontinuously due to failure of the video recorder.

Crayfish		
	Dates Filmed (MM/DD/YYYY)	Total Time Filmed (hr)
1	06/03/2020 – 07/02/2020	44.56
2	07/02/2020 – 7/20/2020	263.82
3	06/28/2020 – 06/30/2020	40.34
4	07/01/2020 – 07/06/2020	105.93
5	06/13/2020 – 06/14/2020; 06/28/2020 – 07/03/20	151.73
6	07/21/2020 – 07/24/2020	57.35

Combined	-	664.73

Ethogram

We reviewed all video footage with Windows Movie Editor Software. While reviewing the footage for each crayfish, we created an ethogram based on observed behaviors (File S1; File S2; File S3). In total, our ethogram contained 5 surface activity behaviors, as well as a single "inactive" behavior (under) which was used to denote when the crayfish was not visible at the surface of the burrow. See Table 2 for detailed descriptions of each behavior. Then, using our ethogram, we recorded what behavior each crayfish was exhibiting, the duration of the behavior, and the time in which the crayfish started and ended the behavior. We also noted whether each crayfish exhibited behaviors during the day or night based on the civil twilight time listed for Hickory, Pennsylvania, U.S.A (collected from www.time.unitarium.com). For our study, we considered a crayfish to be active if they were visible at the surface of their burrow (Fig 3; (35)). We considered a crayfish to be considered inactive if they were not visible at the surface of their burrow. We are unable report on any below-surface activity within our study.

Table 2. Ethogram used to quantify the behaviors from recordings of *Lacunicambarus thomai*.

Behavior	Description
Relax	Crayfish is motionless and partially out of burrow portal with both claws in a "U" orientation directed anteriorly. Movements consist of simple shifts in body position.

Guard	Crayfish is motionless and resting on burrow portal lip (more exposed during the night) with both claws in a “V” orientation. Both claw tips touching or nearly touching directly anterior to the rostrum.
Forage	Crayfish is actively manipulating and foraging for plant material (leaves, grass, or roots).
Excavate	Crayfish is actively carrying, moving, or placing burrow substrate outside of the burrow beyond the burrow portal.
Hunt	Crayfish quickly rushes beyond the burrow portal in attempt to predate on living animal prey.
Under	The crayfish is inactive on the surface and not visible.

Fig 3. Example of relax and guard behaviors from video surveys *of Lacunicambarus thomai*. The top two images (A) and (B) represent crayfish in the relax position, whereas the bottom two image (C) and (D) represent the crayfish in the guard position. Red circles and red lines are presented to give a representation of the differences in articulation between the relax and the guard pose. Photos (A) and (C) were taken at night, whereas photos (B) and (D) were taken during the day.

Environmental data

Because we wanted to investigate broad-scale trends of how the surface activity of *L. thomai* varied with environmental conditions, we gathered environmental data from an online source (www.weatherunderground.com). We collected data from the closest available weather station in

Imperial, Pennsylvania, U.S.A (~24 km away). Specifically, the environmental data that we collected from this source was air pressure, precipitation, temperature, and humidity. Later, these variables were used as covariates to determine which environmental variable best predicted *L. thomai* surface activity.

Statistical analysis

We conducted all statistical analyses in R version 3.5.1 (44). For all of the models described below, we visually inspected our data using the performance package in R to assess normality and collinearity of our model fits (45). For descriptive analyses of *L. thomai* behavior, we calculated the frequency, mean, and range of each behavior. Additionally, we wanted to describe the activity of *L. thomai* based on the time of day. Because of the uneven distribution of video footage length for each crayfish (Table 1), we decided to report the majority of our results in terms of percentages, and not raw values. Therefore, we calculated the percentage of crayfish which exhibited surface activity at each hour by dividing the number of hourly observations in which surface activity was observed by the total number of hours which we recorded throughout our entire study. We averaged these values together for all crayfish which enabled us to investigate patterns in surface activity based on the hour of the day. The same procedure was conducted to determine the percentage of time that each crayfish was observed at the surface throughout the daytime and nighttime.

To determine which environmental variables best related to burrowing crayfish activity, we initially used a model selection procedure with several linear mixed effect models with activity as dependent variable and the environmental variable (i.e., air pressure, precipitation,

temperature, and humidity) as the independent variable. For this analysis, we determined a crayfish to be active during a given hour if they were recorded at the surface of their burrow within any point during that hour. In these models, we included crayfish ID as a random effect. Based on the Akaike information criterion (AICc) of these models, we found that only temperature and humidity effected activity of burrowing crayfish, and therefore we proceeded with these two variables for the remainder of this analysis.

Thus, to evaluate how environmental variables (i.e., humidity and temperature) and time of day (i.e., hour) influenced activity of *L. thomai*, we used multimodal averaging to determine how temperature, humidity, and time (independent variables) influenced the activity of crayfish (dependent variable). For this analysis, we determined a crayfish to be active during a given hour if they were recorded at the burrow portal within any point during the hour. Additionally, because there were multiple observations from each crayfish, we included crayfish ID as a random effect to control for individual differences in individual activity.

We first fit the full model to the data with the *lme4* library of the R statistical package (46). The full model contained temperature, humidity, time, and the interaction between these three variables as the independent variable. Because our dependent variable (i.e., activity) was coded as 1 (active) or 0 (inactive) we used a binomial distribution of error in this model. This model also included crayfish ID as a random variable. Then, we fit models that contained a subset of the terms in the full model. For each model, we calculated the corrected Akaike information criterion and the Akaike weight (i.e., the probability that the model described the data better than the other models in the set). Finally, we used the parameters of each model and Akaike weight to calculate a weighted average of each parameter. These model-averaged parameters enable one to determine the expected probability for any combination of values for

the fixed factors in the full model. This approach eliminates the use of *P* values because all models (including the null model) contributed to the expected probabilities. Importantly, collinearity was unlikely to bias our model averaged parameters, (47); because none of our independent variables (humidity, temperature, and time) were not strongly correlated (all r < 0.6).

To determine whether each specific behavior was more or less likely to occur during the day or night, we conducted six separate Chi-square tests of independence. We conducted 5 separate chi-squared tests for each individual behavior (relax, guard, forage, excavate, hunt, and under). We also performed a single chi-squared test for all six behaviors combined to determine whether all activity was more likely to occur during the day or the night. Additionally, we analyzed the likelihood of each behavior occurring during the day or night with a contingency mosaic plot (48). Continency mosaic plots display the Pearson residual values as colors in relation to the deviation between expected (equally as likely to occur during the day and night) versus the observed frequencies (48). This allows us to both visually and statistically investigate the directionality of the observed frequencies of each behavior (and the combined behaviors) in relation to equal frequencies.

Results

Hourly surface activity

We observed *L. thomai* exhibiting surface activity throughout every single hour of the day. However, there was variation in the times that *L. thomai* were most likely to be observed active at burrow portals. Surface activity peaked from 20:00 hr – 03:00 hr, with nearly every single

crayfish active during those times during the study (Fig 4). After 03:00 hr, crayfish activity slowly declines with surface activity rarely being observed at 17:00hr. Despite being less active throughout the day, 25% to 50% of time each crayfish remained active throughout the daytime.

Fig 4. The percent of *Lacunicambarus thomai* (n=6) that were active for each hour during our study. Gray areas represent the civil twilight time for our study period that represents the transition between day and night. Crayfish were more likely to be active during the night than during the day. Data is reported from all crayfish throughout the entire period of our study.

Environmental predictors of surface activity

Lacunicambarus thomai surface activity was best predicted by a model containing the effect of time, humidity, temperature, an interaction between time and humidity, and an interaction between humidity and temperature (Table 3). However, the three most likely models were all within 2 AICc values of the best fit model (Table 3). Based on the model averaged coefficients, humidity, temperature, and the interaction between humidity and temperature all predicted the activity of *L. thomai* throughout the duration of our study (Table 4). However, the humidity variable had the strongest overall effect (Table 4). Visualizations of the environmental variables are depicted in the Supplemental Informaton (Figure S1, Figure S2, Figures S3, Figure S4).

Table 3. The most likely model predicting the activity of burrowing crayfish based on time, humidity, temperature, an interaction between time and humidity, and an interaction between humidity and temperature. For each model, degrees of freedom (d.f.), the corrected Akaike information criterion (AICc), Akaike weight (*w*), and the log likelihood (ll) are reported.

Models were ranked according to their corrected Akaike information criterion (AICc). Besides the null model, only models with a *w* > 0.00 are reported.

model	d.f.	ll	AICc	DAICc	*w*
Time + humidity + temperature + time*humidity + humidity*temperature	8	-367.90	752.00	0.00	0.42
Time + humidity + temperature + humidity*temperature	7	-369.90	753.96	1.96	0.16
Time + humidity + temperature + time*humidity + time*temperature + humidity*temperature	9	-367.88	754.01	2.00	0.15
Time + humidity + temperature + time*humidity + time*temperature + humidity*temperature + time*humidity*temperature	10	-367.08	754.47	2.47	0.12
Humidity + temperature + humidity*temperature	6	-371.61	755.34	3.34	0.08

Time + humidity + temperature + time*temperature + humidity*temperature	8	-369.70	755.61	3.61	0.07
null	3	-482.07	970.17	218.16	0.00

Table 4. Coefficients and standard errors (SE) for the model predicting the activity of *L. thomai* based on full model averaging. Coefficients are reported in order based on their absolute values. Humidity was the strongest predictor of activity. Thus, the activity of *L. thomai* was negatively related to the degree of environmental humidity.

Independent variable	Coefficient	SE
Humidity	-2.908	1.36
Humidity*temperature	0.057	0.019
Temperature	-0.045	0.012
Time*humidity	0.005	0.089
Time	0.003	0.053
Time*humidity*temperature	< -0.001	0.001
Time*temperature	< 0.001	0.001

Surface activity

Among the six crayfish observed, there was a high degree of surface level activity. Each crayfish spent at least 20% of the time that we filmed at their burrow portal. The percentage of total time each crayfish was observed at the surface ranged from 20.55% (9.18 hr) of the total filmed time to 68.75% (39.42 hr); with an average of 42.48% of the time spent active across all six crayfish (Fig 5). Interestingly, one of our six crayfish (crayfish 6) spent a greater portion of the time active above ground than underground. The remaining 5 *L. thomai* spent a greater percentage of time underground, with a range of 31.25% (17.92 hr) to 79.44% (120.47 hr) of their time spent underground and with an average of 57.52% of each crayfish's time spent underground (Fig 5).

Fig 5. The percentage of time that *Lacunicambarus thomai* spent visible at the surface of their burrow (Active at Surface) or not visible underground in their burrow (Underground). We report the percentage of time that each crayfish individual spent active or inactive as well as a combined total for all six crayfish. For each crayfish, the total number of time (hours) that each crayfish was filmed is listed above each bar.

Day vs. night surface activity

All *L. thomai* exhibited a high degree of surface activity both during the day and during the night. The percentage of time that crayfish were active during the daytime ranged from 20.20% (7.71 hr) to 72.61% (13.65 hr) of the time with an average of 36.46% of activity occurring at the burrow portal or on the surface (Fig 6). Regarding nighttime activity, five of the six crayfish exhibited more nighttime activity at the surface of their burrow than they did daytime activity.

Night activity ranged from 27.38% (5.15 hr) to 79.79% (30.46 hr) with an average of 63.35% of the total surface activity occurring during the night (Fig 6).

Fig 6. The percentage of time that *Lacunicambarus thomai* was observed exhibiting surface activity during the day and during the night. We report the percentage of time that each individual crayfish spent during the day and the night as a combined total for all six crayfish. For each crayfish, the total number of time (hours) that each crayfish was filmed active is listed above the bar.

The behaviors exhibited by *L. thomai* varied depending on whether they were active during the day or night (Table 5). The most common diurnal behavior recorded was the guard and relax behavior, which comprised nearly 80% of the total time crayfish were observed at the surface during the day (Fig 7). Interestingly, a single crayfish (crayfish 4) exhibited foraging behavior during the day for 3.9% (0.34 hr) of the time that they were filmed. We observed two crayfish (crayfish 1 and 5) excavating their burrow during the day. Overall, each crayfish spent most of their day activity exhibiting the guard behavior at their burrow portal (Fig 3; Table 5).

Table 5. The number of observations and the mean duration of each behavior observed during our study. Each behavior is split based on whether the behavior occurred during the day or night. Data is reported based on the mean duration of each behavior from all six crayfish in our study.

Behavior	Day		Night	
	No. observations	Mean duration	No. observations	Mean duration

Relax	63	648 s	320	1116 s
Guard	183	9468 s	134	792 s
Forage	1	360 s	227	432 s
Excavation	10	102.4 s	199	166.39 s
Hunt	3	120.33 s	29	80.72 s
Under	260	4572 s	509	504 s

Fig 7. The percentage of time that each *Lacunicambarus thomai* spent exhibiting different behaviors throughout the (A) day and the (B) night. We report the percentage of time that each individual crayfish spent during the day and the night and combined six crayfish. The hunt behavior was not included in because of the relatively small time that this behavior was exhibited. For each crayfish, the total number of time (hours) that each crayfish was filmed active is listed above the bar.

Crayfish showed a greater level of nocturnal surface activity. The majority of their time was spent in the relax position during the night, and guarding was more likely to occur during the night compared to during the day (Fig 7); both of these behaviors occurred at burrow portals.

Interestingly, each crayfish spent a portion of their nighttime activity exhibiting foraging behavior, with an average of 10.5% of all night activity including foraging (Fig 7). The same two crayfish that excavated during the day (crayfish 1 and crayfish 5) were also observed excavating their burrow at night, but they excavated for a greater percentage of their nighttime activity than their daytime activity (Fig 7).

When analyzing the likelihood of each behavior occurring at day versus night, the chi-squared analyses demonstrated that every behavior was more likely to occur during one light period compared to another. Specifically, the guard behavior and inactivity (i.e., under) were more likely to occur during the day compared to the night (Fig 8; Table 6). By contrast, excavate, forage, and the relax behavior were more likely to occur at night (Fig 8; Table 6). The hunt behavior was also more likely to occur during the night than during the day (Fig 8; Table 6).

Fig 8. Mosaic plot of the behavior exhibited by each crayfish (excavate, forage, guard, hunt, relax, under) based on the time of day it was recorded (night, day). The width of the bars represents the number of observations that we recorded, whereas the height of the bars represents the number of observations that were recorded in the night (the bottom bar) versus the day (the top bar). The color of each bar represents whether the observed frequency of each behavior deviates from the expected frequencies if the variables were independent. Red denotes observed frequencies that are smaller than the expected frequency, whereas blue represents the observed frequencies that are larger than the expected frequencies. Gray represents a similar observed frequency when compared to the expected frequency.

Table 6. Results from Chi-squared test for the number of behaviors that occur during the day and versus the night. For each behavior, we report the degrees of freedom (d.f.), Chi-squared value ($\chi 2$), and *p* value. All behaviors showed significant differences between the number of times the behavior was observed during the day or night.

Behavior			
	d.f.	$\chi 2$	*p*
Relaxed	1	178.94	0.002
Guard	1	15.01	< 0.001
Forage	1	171.07	< 0.001
Excavate	1	92.45	< 0.001
Hunt	1	9.32	< 0.001
Under	1	66.16	< 0.001
All behaviors	5	269.29	< 0.001

Discussion

Out of the 664.73 hrs that we observed across six *L. thomai*; crayfish were active at the surface of their burrow nearly half of this time. Furthermore, we observed a surprisingly high amount of surface activity during the day, with nearly one-third of all surface activity occurring during the day and not at night. With our footage, we were also able to create an ethogram based on the five different surface behaviors (relaxed, guard, forage, excavate, and hunt) that we observed. Our ethogram allowed us to examine the behavioral repertoire and prevalence of the whether the observed behaviors more frequently occurred during the day or night. We discuss these findings

in relation to previously published observations of burrowing crayfish surface behavior as well as potential opportunities for future research.

Before our study, little has been reported on the degree of surface activity and specific behaviors exhibited by burrowing crayfishes. Although surface activity has been observed throughout burrowing crayfishes both during the day and during the night, the prevalence in which *L. thomai* was active at the surface far exceeds that of other previously published reports. Previous accounts suggest that burrowing crayfish were only active above ground during a few instances which included high water events (50), during cloudy days according to light levels (35), or when looking for food or mates (12). However, many of these accounts come from *in situ* natural history observations, and not systematic investigations. For example, Loughman *et al*. 2015 reports over 50 observations of *Cambarus pauleyi* at the surface of their burrow, but only three of these observations were made during the day (51). Moreover, Foltz *et al*. 2018 states that *Cambarus loughmani* did not rise from burrows until a few hours after twilight, with no daylight activity reported (26).

Although we studied a small number of *L. thomai* during a two-month summer period, every crayfish was active during peak activity times. Only two previous studies have reported hourly activity data for burrowing crayfishes (34,37). For over a year, Bearden *et al*. (2021) collected hourly activity data for two burrowing crayfish species (*Lacunicambarus erythrodactylus* and *Procambarus holifieldi)*. In their study, both *L. erythrodactylus* and *P. holifieldi* were observed active roughly 25% of the time during the night and were rarely observed being active during daytime hours (37). In a laboratory study, Palaoro *et al*. 2013 collected hourly data on a South American burrowing species (*Parastacus brasiliensis*) and

showed that this species spent a majority of their experiment within their burrow, but were more likely to leave their burrow during the night compared to during the day (34).

In our study, the high degree of surface activity observed may be specific to crayfish in the genus *Lacunicambarus*. However, because our study was conducted on few individuals, future research on the surface behavior of *Lacunicambarus thomai* and the other 13 species in the genus *Lacunicambarus* is warranted (52). Alternatively, other burrowing species (and genera) may also be highly active during the day, but researchers often exclusively collect burrowing crayfish during the night which can bias the prevalence of these behavioral observations (24,53). For example, in a seven year-long ecological study of the Digger Crayfish, *Creaserinus fodiens*, Norrocky *et al*. 1991 reported that all crayfish collections occurred at night (54). Clearly, the high degree of surface activity that we found in *L. thomai* contrasts our previous understanding of the activity and surface behaviors of burrowing crayfishes. Further, our study solely reports observations of surface behavior, and we are unable to report on the presence, absence, or degree of underground activity.

Based on our analysis of the environmental predictors of surface activity, we found that humidity had the strongest overall effect of predicting burrowing crayfish surface activity. Specifically, we found that crayfish were more likely to be active at lower humidity values. These results contrast previous findings by Bearden et al. 2021, in which activity was best predicted by a combination of variables, including daylength, air temperature, and groundwater temperature. Importantly, our analysis only uses reported data from the macro-environment at a nearby weather station, and not local, micro-environmental variables (variables recorded at the exact environment of or study site), as was done by Bearden and colleagues (37). For example, in their study, Bearden et al. (2021) found that chimney constructed was related to precipitation

events. It remains unknown whether the other surface behaviors which we describe have similar correlations to environmental variables such as prescription or groundwater temperature. Future studies should take into account the variation within environmental variables such as air temperature and burrow temperature to investigate behavioral patterns in burrowing crayfish. For example, activity in burrowing mammals is often best predicted by an interaction between both surface and groundwater temperatures (55). Therefore, similar surface and groundwater temperature interactions may occur in burrowing crayfishes which are already known to use their burrows as refuges from harsh environmental temperatures (12,13,37).

Besides our study, only one other study has created an ethogram specific to burrowing crayfish surface behavior, although this study was conducted in a laboratory environment (34). By contrast, our *in-situ* recordings allowed us to create an ethogram based entirely on natural behaviors, which revealed the richness of burrowing crayfish surface behavior—many of which have been previously undocumented. In our study, because *L. thomai* were constantly being monitored, we were able to categorize each crayfish into one of five different surface behaviors. The two most prevalent activities were the relax behavior and the guard behavior, which we separated based on the orientation of the crayfish's claws relative to their body position (Fig 3). Whether the guard behavior's function is truly to defend the burrow is unknown, because we did not observe any intraspecific interactions in our study. However, defense, guarding-like behavior has been reported in other burrowing crayfish (51).

Interestingly, there were strong differences in the prevalence of the guard and relax behaviors depending on whether it was during the day or night. We found that during the day, *L. thomai* was more likely to be active in the guarded position compared to the relax position. By contrast, when we observed *L. thomai* active during the night, they were much more likely to be

in the relax position than the guard position. The function of these different postural behaviors is unknown and requires further investigation. But we can speculate that the guard behavior is more likely to be exhibited during the day due to the increased potential for predator interactions. Indeed, we observed terrestrial predators (i.e., snakes and birds) interacting with *L. thomai* burrows during the day (36). Alternatively, the rest behavior being more likely to occur during the night may relate to this posture's increased ability to quickly capture live prey that move near the burrow (35,36,41) . Future studies must investigate whether or not these postural differences are present throughout other burrowing crayfishes, or if they are specific to *L. thomai*.

We also observed 271 instances of *L. thomai* engaging in active foraging for nearby vegetation. Within our observations, *L. thomai* often explored the area within the vicinity of their burrow portal and used their claws to cut nearby vegetation. Across all our observations, only a single instance of vegetation foraging occurred during the daytime, which demonstrates a strong preference for this behavior occurring at night when there are presumably fewer active predators. Interestingly, the vegetation was either immediately consumed at the base of the burrow or was taken down into the burrow out of the view of the camera. Therefore, our observations confirmed previous reports of burrowing crayfish foraging; because the area surrounding the burrow portals are often replete with vegetation (26). Furthermore, crayfish burrows are commonly excavated into large chambers, and large amounts of vegetation are found within the burrow tunnels and the burrow chambers, which are likely to serve as a food storage cache (26,27,56). Although our observations confirm that *L. thomai* does take vegetation down into the burrow it is difficult to confirm the hypothesis that *L. thomai* burrows may serve as a food cache. The use of crayfish burrowing chambers in laboratory settings (29) is required to confirm the possibility for crayfish using their burrows to store and consume food resources.

In addition to observations of *L. thomai* foraging for vegetation, we observed 32 instances of the crayfish exhibiting sit and wait hunting behavior. In these observations, the crayfish sat at the burrow portal and quickly lunged at live animal prey (i.e., the hunt behavior). In some instances, this ambush behavior successfully resulted in the capture of live animal prey, whereas other attempts did not result in successful acquisition of the prey. When this behavior resulted in a successful attempt, the crayfish immediately took the item into their burrow. This ambush behavior was more likely to occur nocturnally than diurnally, with only three of 32 observations occurring during the day. Interestingly, this sit and wait predatory behavior seems to be prevalent throughout the genus *Lacunicambarus*, as it has been reported in at least 2 other *Lacunicambarus* species (35,36,41). Recently, Thoma (2022) reports that in Ohio, *Lacunicambarus chimera* is never active at the surface of their borrow, whereas another species, *Lacunicambarus nebrascensis* rarely exhibits surface activity (57). Although other species of crayfish have been observed capturing live animal prey (28,58), the degree and reliance on this ambush predation behavior across burrowing crayfishes is unknown.

Across the six crayfish that we studied, we only observed two *L. thomai* actively excavating their burrows. These observations indicate burrow excavation is not an exceedingly common *L. thomai* behavior (34,37), and may only occur when necessary alterations to the burrow architecture are needed. Each observation of excavation behavior always occurred in a repeated series of ritualized behaviors. First, crayfish would be observed bringing mud up to the burrow portal. Once at the surface, the crayfish would immediately stop before reaching ground level and immediately tap their antennae at their edge of their burrow portal. Lastly, the crayfish would finally emerge and place the mud pellet and methodically use its claws the push the mud into position.

In previous studies of *P. holifieldi,* precipitation was one of the variables that influenced the likelihood of burrow excavation (i.e., referred to as chimney construction in the study) (37). Indeed, burrows become flooded during times of rainfall and often require renovations following these events (Z. Loughman, personal observations). Only two small rain events occurred during our study period, which may explain the lack of burrow excavation being observed. Furthermore, excavation behavior was more likely to occur at night than during the day. Intuitively, burrow excavation is a relatively costly behavior, given substrates must repeatedly be excavated from the bottom of the burrow to the surface. Such behaviors are more likely to have increased costs during the day, both based on the high surface temperatures and the potential for increased rates of predation during the daytime.

In summary, based on our preliminary investigation, we found that *L. thomai* exhibits a high degree of surface activity. Further, we found that *L. thomai* exhibits several behaviors at ~~the~~ their burrow portal, and these behaviors all were more likely to occur during either day or night. However, our study brings several unanswered questions to light regarding the behavior of burrowing crayfishes. For example, although we observed many small foraging excursions that occurred within the immediate vicinity of the burrow, we did not observe a single large-scale excursion during this time. In other species of burrowing crayfishes, substantial surface activity occurs immediately after rainfall, and such crayfish may travel large distances in a short period of time (12; Z. Loughman & Z. Graham personal observations). Although the exact function of these excursions is unknown, it is unclear whether or not *L. thomai* engages in such behaviors.

Further, throughout our study, we did not observe any intra-specific interactions. Again, scattered observations highlight the social behavior of burrowing crayfishes, but interestingly, we did not observe any social behavior throughout the course of our study. Future studies should

conduct similar behavioral observations throughout the year and also investigate potential demographic differences in the behaviors we report. By expanding the time frame in which records occur, social interactions of larger foraging excursions may be recorded. Lastly, although our study has shed light on the activity of crayfishes at the surface, much remains unknown about the subsurface behavior of burrowing crayfishes. Additionally, since we discovered that this species displayed easily identifiable postural behaviors, this may allow for automated video analysis of body position, activity, and behaviors. As such, the continued use of burrowing crayfish observations chambers (29) will be paramount in furthering our understanding of the behavioral ecology of burrowing crayfishes. In conclusion, our study revealed a surprisingly high degree of surface activity in a species of burrowing crayfish. Based on the historical difficulties in studying burrowing crayfish, there is much to learn about the ecological and evolutionary dynamics of burrowing crayfish behavior.

Acknowledgements

We thank the Davis family for the use of their land for the study location.

Author Contributions

Conceived and designed the study: Z.J.L, D.A.D, K.M.D, Z.A.G. Collected the data: K.M.D D.A.D, H.D.W. Analyzed the data: K.M.D, Z.A.G. All authors contributed to writing the paper.

References

1. Richman NI, Böhm M, Adams SB, Alvarez F, Bergey EA, Bunn JJS, et al. Multiple drivers of decline in the global status of freshwater crayfish (Decapoda: Astacidea). Philos Trans R Soc B Biol Sci. 2015;370(1662):1–11.

2. Crandall KA, DeGrave S. An updated classification of the freshwater crayfishes (Decapoda: Astacidea) of the world, with a complete species list. J Crustac Biol. 2017;37(5):615–53.

3. Moore P. Agonistic behavior in freshwater crayfish: the influence of intrinsic and extrinsic factors on aggressive behavior and dominance. Evol Ecol Soc Sex Syst Crustac as Model Org. 2007;90–114.

4. Jimenez S, Faulkes Z. Establishment and care of a laboratory colony of parthenogenetic Marbled Crayfish, Marmorkrebs. Invertebr Rearing [Internet]. 2010;1(1):10–8. Available from: http://inverts.info/content/establishment-and-care-laboratory-colony-parthenogenetic-marbled-crayfish-marmorkrebs

5. Seitz R, Vilpoux K, Hopp U, Harzsch S, Maier G. Ontogeny of the Marmorkrebs (marbled crayfish): A parthenogenetic crayfish with unknown origin and phylogenetic position. J Exp Zool Part A Comp Exp Biol. 2005;303(5):393–405.

6. Reynolds J, Souty-Grosset C. Management of freshwater biodiversity: Crayfish as bioindicators. Management of Freshwater Biodiversity: Crayfish as Bioindicators. 2011. 1–388 p.

7. Herberholz J, Antonsen BL, Edwards DH. A lateral excitatory network in the escape

circuit of crayfish. J Neurosci. 2002;22(20):9078–85.

8. Stern DB, Breinholt J, Pedraza-Lara C, López-Mejía M, Owen CL, Bracken-Grissom H, et al. Phylogenetic evidence from freshwater crayfishes that cave adaptation is not an evolutionary dead-end. Evolution (N Y). 2017;71(10):2522–32.

9. Breinholt JW, Porter ML, Crandall KA. Testing Phylogenetic Hypotheses of the Subgenera of the Freshwater Crayfish Genus Cambarus (Decapoda: Cambaridae). PLoS One [Internet]. 2012 [cited 2019 Feb 5];e46105. Available from: www.plosone.org

10. Moore MJ, DiStefano RJ, Larson ER. An assessment of life-history studies for USA and Canadian crayfishes: identifying biases and knowledge gaps to improve conservation and management. Freshw Sci [Internet]. 2013;32(4):1276–87. Available from: http://www.journals.uchicago.edu/doi/10.1899/12-158.1

11. Berrill M, Chenoweth B. The Burrowing Ability of Nonburrowing Crayfish. Am Midl Nat. 1982;108(1):199–201.

12. Hobbs HH. The Crayfishes of Georgia. Smithson Contrib to Zool. 1981;(318):1–549.

13. Richardson AMM. Behvioral ecology of semterrestrial crayfish. In: Evolutionary ecology of social and sexual systems: Crustacae of model organisms. 2007. p. 219–338.

14. Hobbs HH. The crayfishes of Florida. Univ Florida Publicaiton, Biol Sci Ser. 1942;3(2):1–179.

15. Pintor LM, Soluk DA. Evaluating the non-consumptive, positive effects of a predator in the persistence of an endangered species. Biol Conserv. 2006;130(4):584–91.

16. Holycross AT, Mackessy SP. Variation in the Diet of Sistrurus catenatus (Massasauga),

with Emphasis on Sistrurus catenatus edwardsii (Desert Massasauga) Author (s): Andrew T . Holycross and Stephen P . Mackessy Published by : Society for the Study of Amphibians and Reptiles S. J Herpetol. 2002;36(3):454–64.

17. Krupa JJ, Hopper KR, Nguyen MA. Dependence of the dwarf sundew (Drosera brevifolia) on burrowing crayfish disturbance. Plant Ecol [Internet]. 2021;222(4):459–67. Available from: https://doi.org/10.1007/s11258-021-01119-3

18. Creaser EP. Some cohabitants of burrowing crayfish. Ecology. 1931;12(1):243–4.

19. Welch SM, Waldron JL, Eversole AG, Simoes JC. Seasonal variation and ecological effects of camp shelby burrowing crayfish (Fallicambarus gordoni) burrows. Am Midl Nat. 2008;159(2):378–84.

20. Reynolds J, Souty-Grosset C, Richardson A. Ecological roles of crayfish in freshwater and terrestrial habitats. Freshw Crayfish. 2013;19(2):197–218.

21. Richardson AMM. The effect of the burrows of a crayfish on the respiration of the surrounding soil. Soil Biol Biochem. 1983;15(3):239–42.

22. Graham ZA, Angilletta MJ. Distinguishing between unreliability and dishonesty: A comparitive study of aggressive communciation in crayfish. Funct Ecol. 2021;

23. Welch SM, Eversole AG. Comparison of Two Burrowing Crayfish Trapping Methods. Southeast Nat. 2016;5(1):27–30.

24. Ridge J, Simon TP, Karns D, Robb J, Fish US, Service W, et al. Comparison of three burrowing cray- fish capture methods based on relationships with species morphology, seasonality, and habitat quality. J Crustac Biol. 2008;28(3):466–72.

25. Glon MG, Thoma RF. An observation of the use of devil crayfish (Cambarus cf. diogenes) burrows as brooding habitat by Eastern cicada killer wasps (Sphecius speciosus). Freshw Crayfish. 2017;23(1):55–7.

26. Foltz DA, Sadecky NM, Myers GA, Fetzner JW, Welsh SA, Stocker GW, et al. Cambarus loughmani, a new species of crayfish (Decapoda: Cambaridae) endemic to the pre-glacial Teays River Valley in West Virginia, USA. J Nat Hist [Internet]. 2018;52(45–46):2875–97. Available from: https://doi.org/10.1080/00222933.2018.1557271

27. Loughman ZJ, Welsh SA, Thoma RF. Cambarus fetzneri sp. nov., a new species of burrowing crayfish (Decapoda: Cambaridae) from the Allegheny Mountains of Virginia and West Virginia, USA. Zootaxa. 2019;4651(1):38–50.

28. Loughman ZJ. Ecology of Cambarus dubius (Upland Burrowing Crayfish) in North-central West Virginia. Southeast Nat. 2010;9(SPECIAL ISSUE 3):217–30.

29. Stoeckel JA, Helms BS, Cash E. Evaluation of a crayfish burrowing chamber design with simulated groundwater flow. J Crustac Biol. 2011;31(1):50–8.

30. Clay M, Stoeckel J, Helms B. The role of abiotic and biotic cues in burrow habitat selection by juvenile crayfish. Behaviour. 2017;154(12):1177–96.

31. Dalosto MM, Palaoro A V, Costa JR, Santos S. Aggressiveness and life underground : the case of burrowing crayfish. Behavior. 2013;150:3–22.

32. Costa JR, Dalosto MM, Palaoro A V, Santos S. Contest Duration and Dynamics are Affected by Body Size in a Potentially Subsocial Crayfish (Crustacea : Decapoda). 2016;122:502–12.

33. Grow L. Burrowing behaviour in the crayfish, Cambarus diogenes diogenes girard. Anim Behav. 1981;29(2):351–6.

34. Palaoro A V., Dalosto MM, Coutinho C, Santos S. Assessing the importance of burrows through behavioral observations of parastacus brasiliensis, a neotropical burrowing crayfish (crustacea), in laboratory conditions. Zool Stud. 2013;52(1):1–8.

35. Thoma RF, Armitage BJ. Burrowing Crayfish of Indiana. 2008;1–226.

36. Graham ZA, Diehl KM, Davis D, Loughman ZJ. Death from below : Sit-and-wait predatory behavior in a burrowing crayfish (Lacunicambarus thomai). Food Webs [Internet]. 2022;31(January):e00225. Available from: https://doi.org/10.1016/j.fooweb.2022.e00225

37. Bearden RA, Tompkins EM, Huryn AD, Schuster P. Motion-triggered laser photography reveals fine- - scale activity patterns for burrowing crayfish. 2021;(January):1–13.

38. Florey CL, Moore PA. Analysis and description of burrow structure in four species of freshwater crayfishes (Decapoda: Astacoidea: Cambaridae) using photogrammetry to recreate casts as 3D models. J Crustac Biol. 2019;39(6):711–9.

39. Loughman ZJ, Simon TP. Zoogeography, taxonomy, and conservation of West Virginia’s Ohio River floodplain crayfishes (Decapoda, Cambaridae). Zookeys. 2011;74(SPEC. ISSUE):1–78.

40. Loughman ZJ. Crayfishes of western Maryland: Conservation and natural history. Southeast Nat. 2010;9(SPECIAL ISSUE 3):33–62.

41. Simon TP, Morris CC. Cambarus (Lacunicambarus) erythrodactylus, a new species of

crayfish (Decapoda: Cambaridae) of the Cambarus diogenes complex from Alabama and Mississippi, U.S.A. Proc Biol Soc Washingt. 2015;127(4):572–84.

42. Glon MG, Adams SB, Loughman ZJ, Myers GA, Taylor CA, Schuster GA. Two new species of burrowing crayfish in the genus Lacunicambarus (Decapoda: Cambaridae) from Alabama and Mississippi. Vol. 4802, Zootaxa. 2020. 401–439 p.

43. Miller JM, Niraula BB, Reátegui-Zirena EG, Stewart PM. Life history and physical observations of primary burrowing crayfish (Decapoda: Cambaridae) Cambarus (Lacunicambarus) diogenes and Cambarus (Tubericambarus) polychromatus. J Crustac Biol. 2014;34(1):15–24.

44. R Core Team: A Lang Environ Stat Comput. 2019;(R Foundat.

45. Lüdecke D, Ben-Shachar MS, Patil I, Waggoner P, Makowski D. performance: An R package for assessment, comparison and testing of statistical models. J Open Source Softwater. 2021;6.60.

46. Bates DM. lme4: Mixed-effects modeling with R. 2010;

47. Cade BS. Model Averaging and Muddled Multimodel Inferences. Ecology. 2015;96(9):2370–82.

48. Meyer D, Zeileis A, Hornik K. The strucplot framework: Visualizing multi-way contingency tables with vcd. J Stat Softw. 2006;17(3):1–48.

49. Burnham KP, Anderson DR. Model Selection and Multimodel Inference: A Practical Information-Theoretic Approach. Springer; 2002.

50. Norrocky JM. Observations on the Ecology , Reproduction and Growth of the Burrowing

Crayfish Fallicambarus (Creaserinus) fodiens (Decapoda : Cambaridae) in North-central Ohio. Am Midl Nat. 1991;125(1):75–86.

51. Loughman ZJ, Thoma RF, Fetzner JW, Stocker GW. Cambarus (Jugicambarus) pauleyi, a new species of crayfish (Decapoda: Cambaridae) endemic to southcentral West Virginia, USA, with a re-description of Cambarus (J.) dubius. Zootaxa. 2015;3980(4):526–46.

52. Hobbs L, Astacidea D, Glon MG, Broe MB, Crandall KA, Daly M, et al. Anchored hybrid enrichment resolves the phylogeny of Lacunicambarus Hobbs, 1969 (Decapoda: Astacidea: Cambaridae). 2022;42(January):1–18.

53. Loughman ZJ, Foltz DA, Welsh SA. Baited lines: An active nondestructive collection method for burrowing crayfish. Southeast Nat. 2013;12(4):809–15.

54. Norrocky MJ. Observations on the Ecology, Reproduction and Growth of the Burrowing Crayfish Fallicambarus (Creaserinus) fodiens (Decapoda: Cambaridae) in North-central Ohio. Am Midl Nat. 1991;125(1):75.

55. Jannetti MG, Buck CL, Valentinuzzi VS, Oda GA. Day and night in the subterranean: Measuring daily activity patterns of subterranean rodents (Ctenomys aff. knighti) using bio-logging. Conserv Physiol. 2019;7(1):1–13.

56. Jezerinac RF, Stocker GW, Tarter DC. Crayfishes (Decapoda: Cambaridae) of West Virginia. Ohio Biological Survey,College of Biological Sciences, the Ohio State University; 1995.

57. Thoma RF. A Naturalist's Guide to the Crayfish of Ohio. Ohio Biological Survey; 2022.

58. Mccormack RB, Coughran J. Taxonomy, distribution and ecology of the Setose Yabby,

Cherax setosus (Riek, 1951). Crustac Res. 2011;40(January):1–11.

Supporting information

S1 File. Recording of an excavation observation during the night.

S2 File. Recording of a foraging observation during the night.

S3 File. Recording of a hunt observation during the night.

S1 Figure. Average, Minimum, and Maximum Air Temperature at our study location in Hickory, PA throughout our study period (June 2020 – July 2020).

S2 Figure. Average, Minimum, and Maximum Humidity at our study location in Hickory, PA throughout our study period (June 2020 – July 2020).

S3 Figure. Average, Minimum, and Maximum Air Pressure at our study location in Hickory, PA throughout our study period (June 2020 – July 2020).

S4 Figure. Precipitation at our study location in Hickory, PA throughout our study period (June 2020 – July 2020).

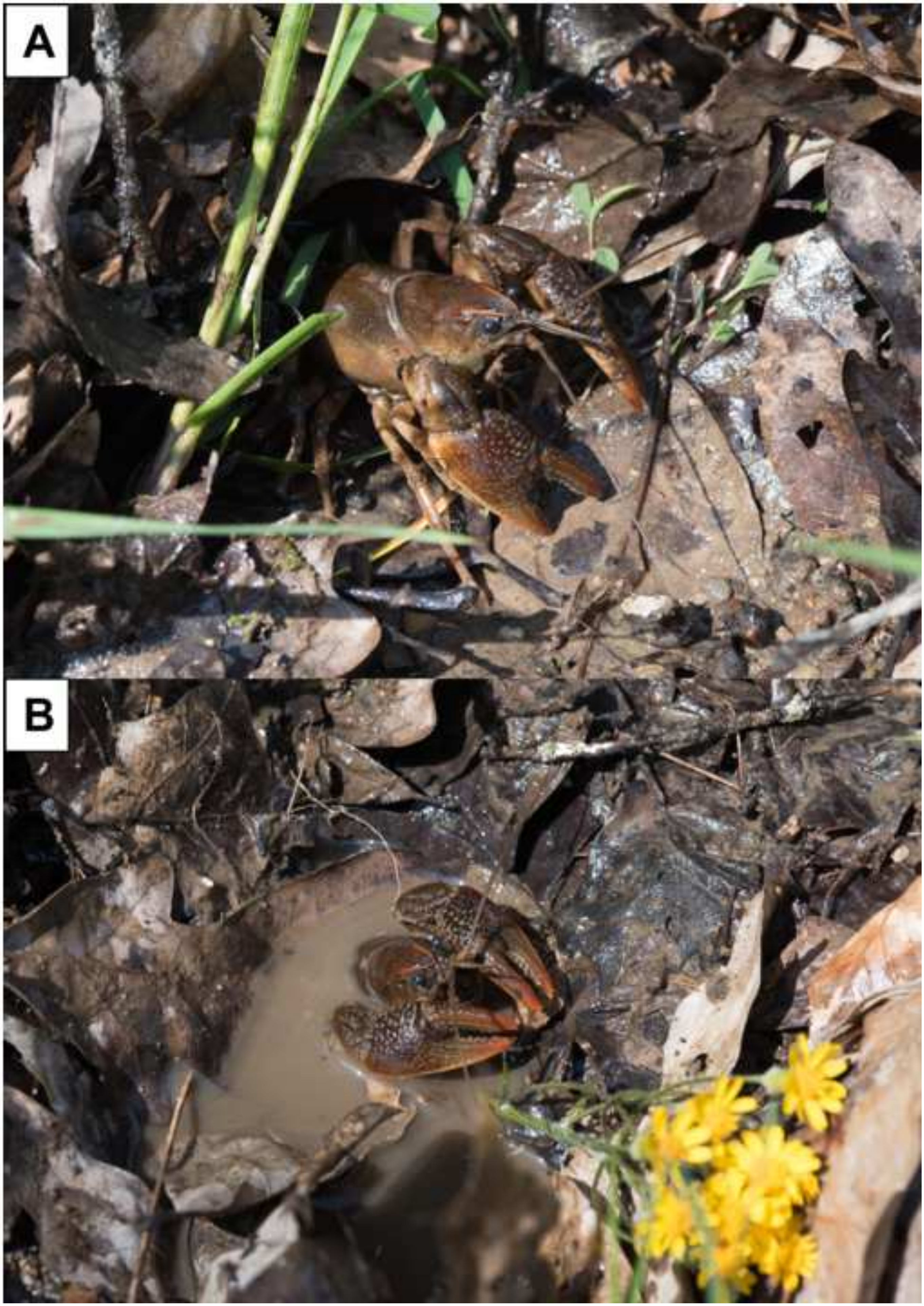
A
B

Figure 2

Click here to access/download;Figure;Fig2.tif

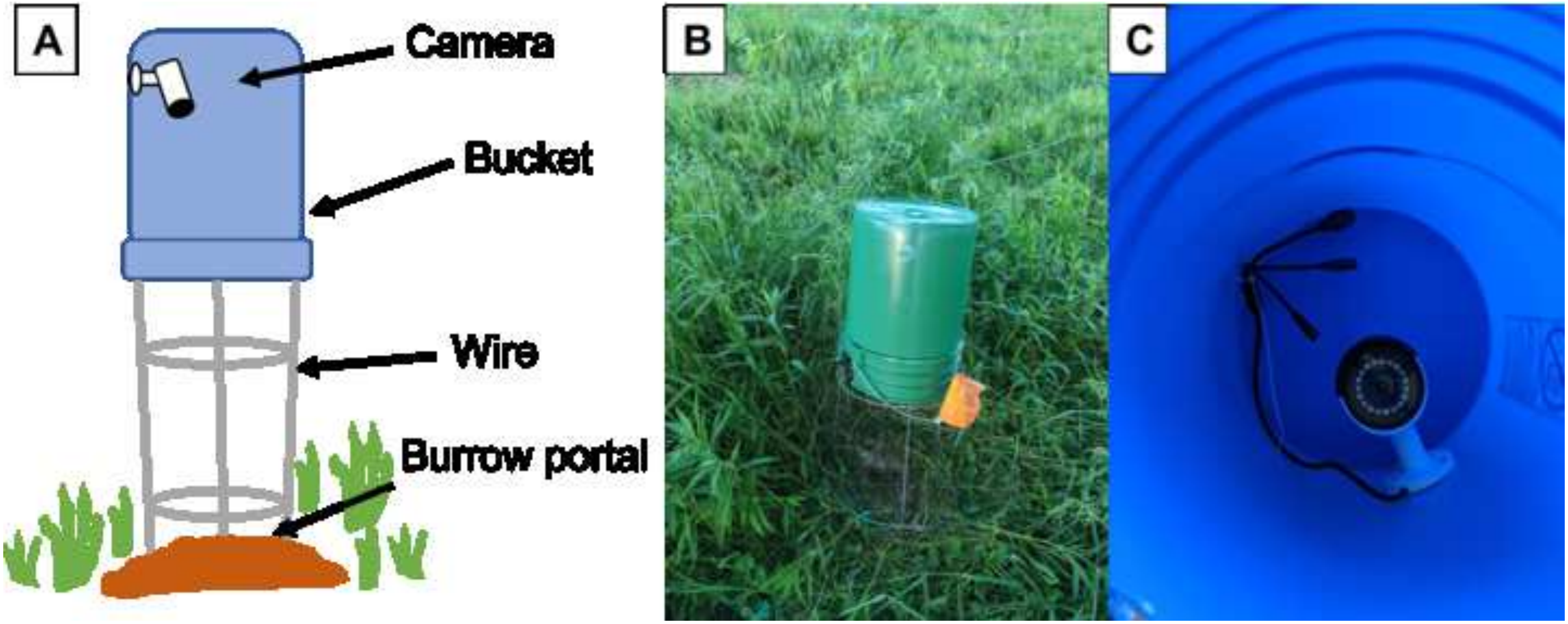

Figure 3

Click here to access/download;Figure;Fig3.tif

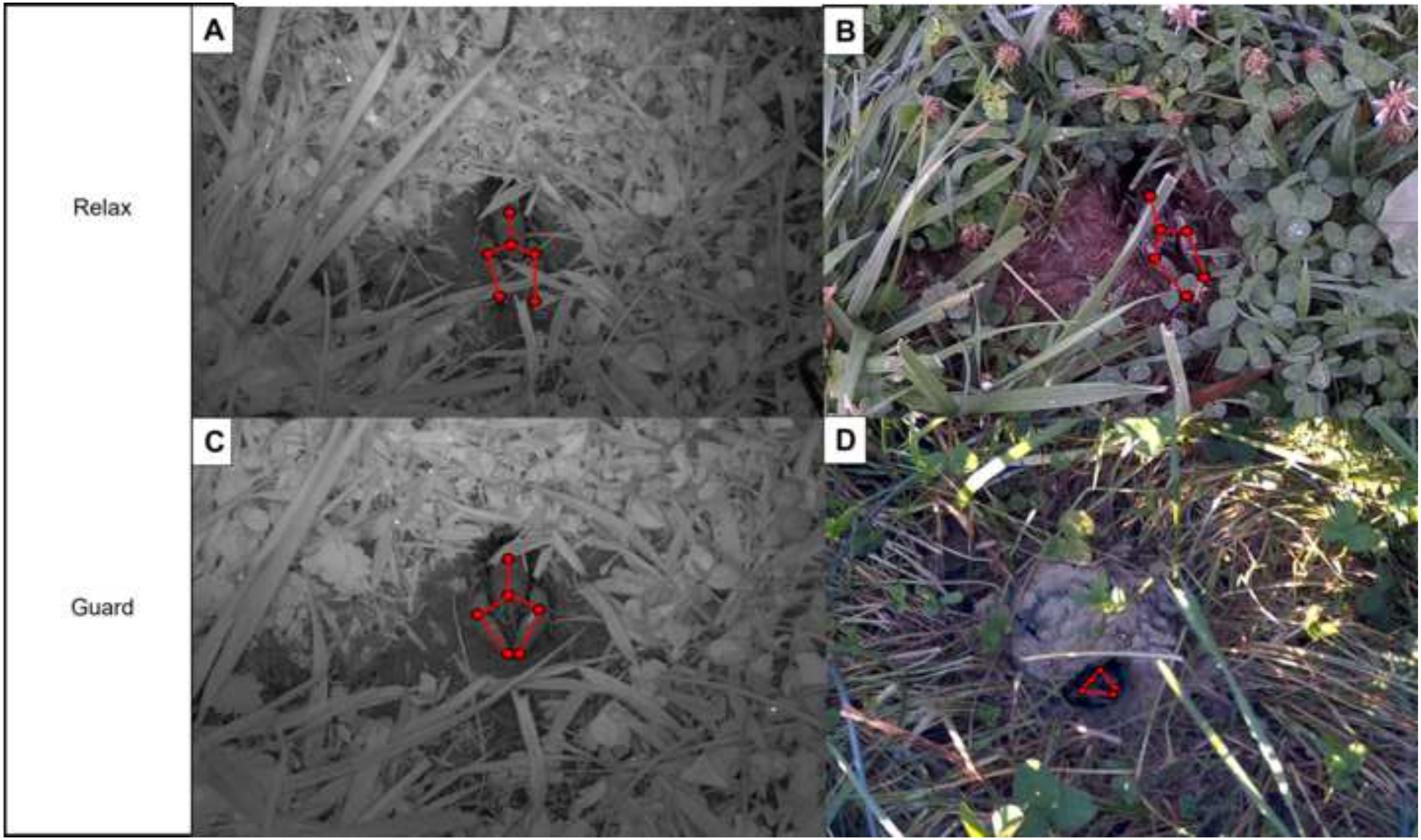

Figure 4

Click here to access/download;Figure;Fig4.tif

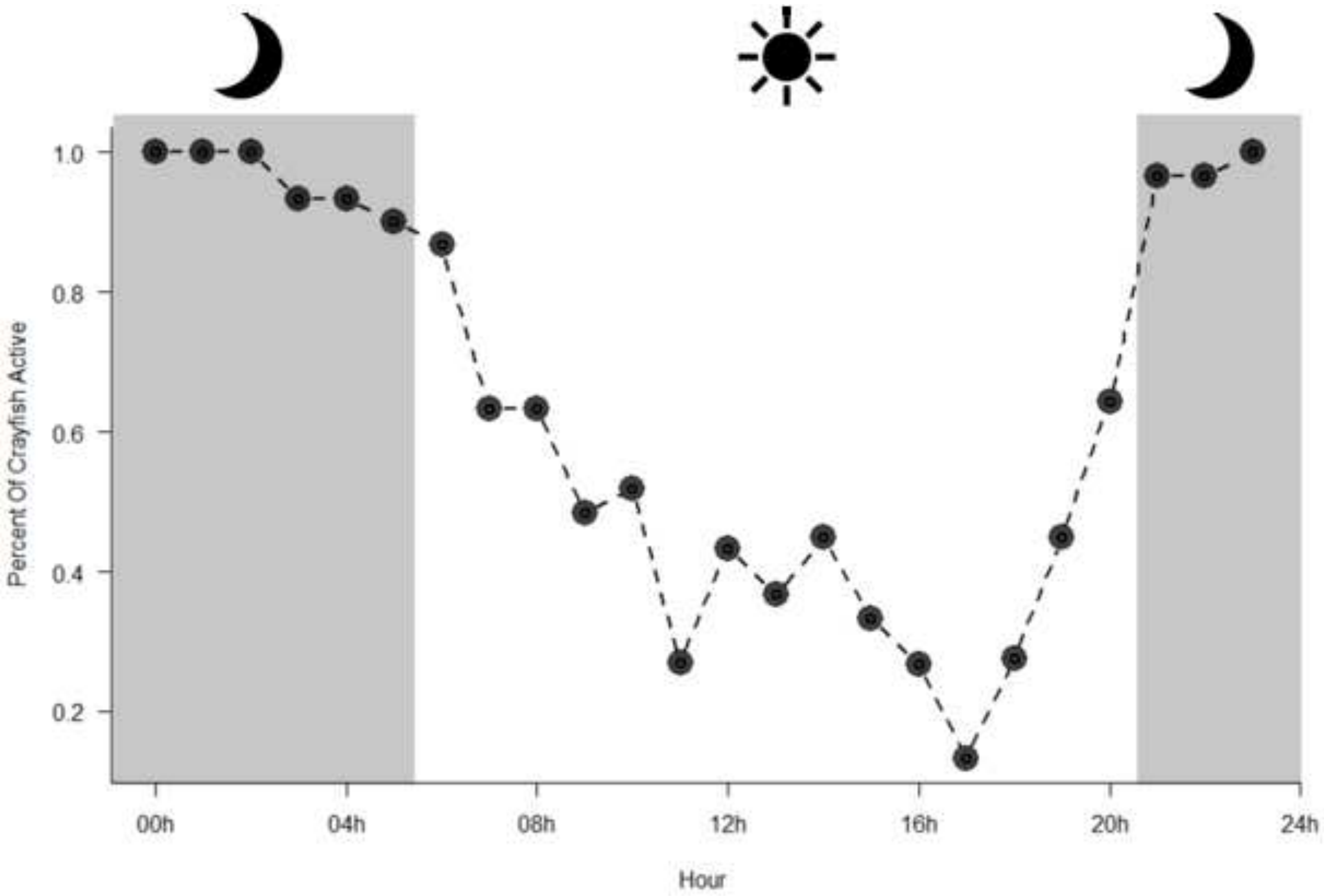

Figure 5

Click here to access/download;Figure;Fig5.tif

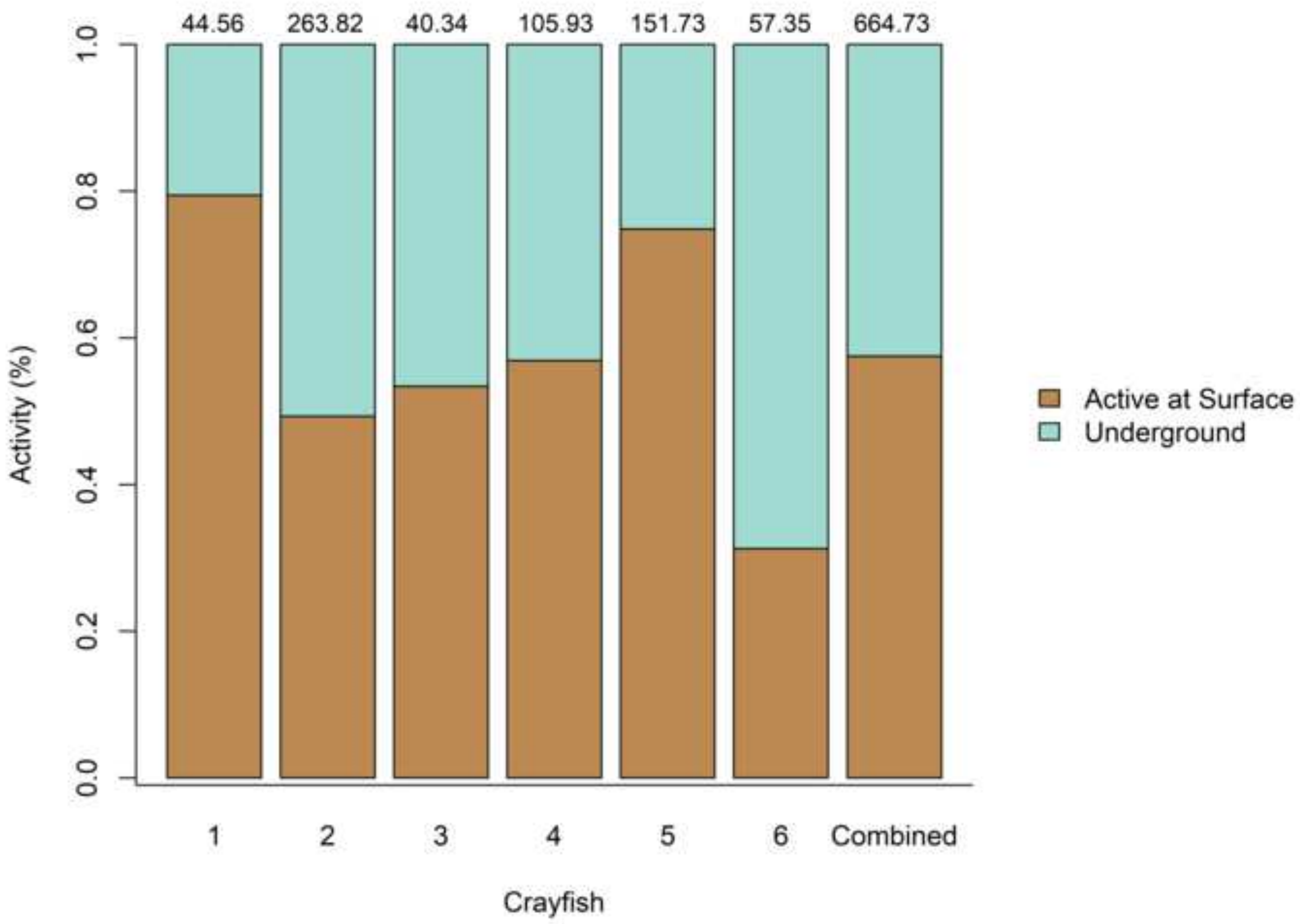

Figure 6

Click here to access/download;Figure;Fig6.tiff

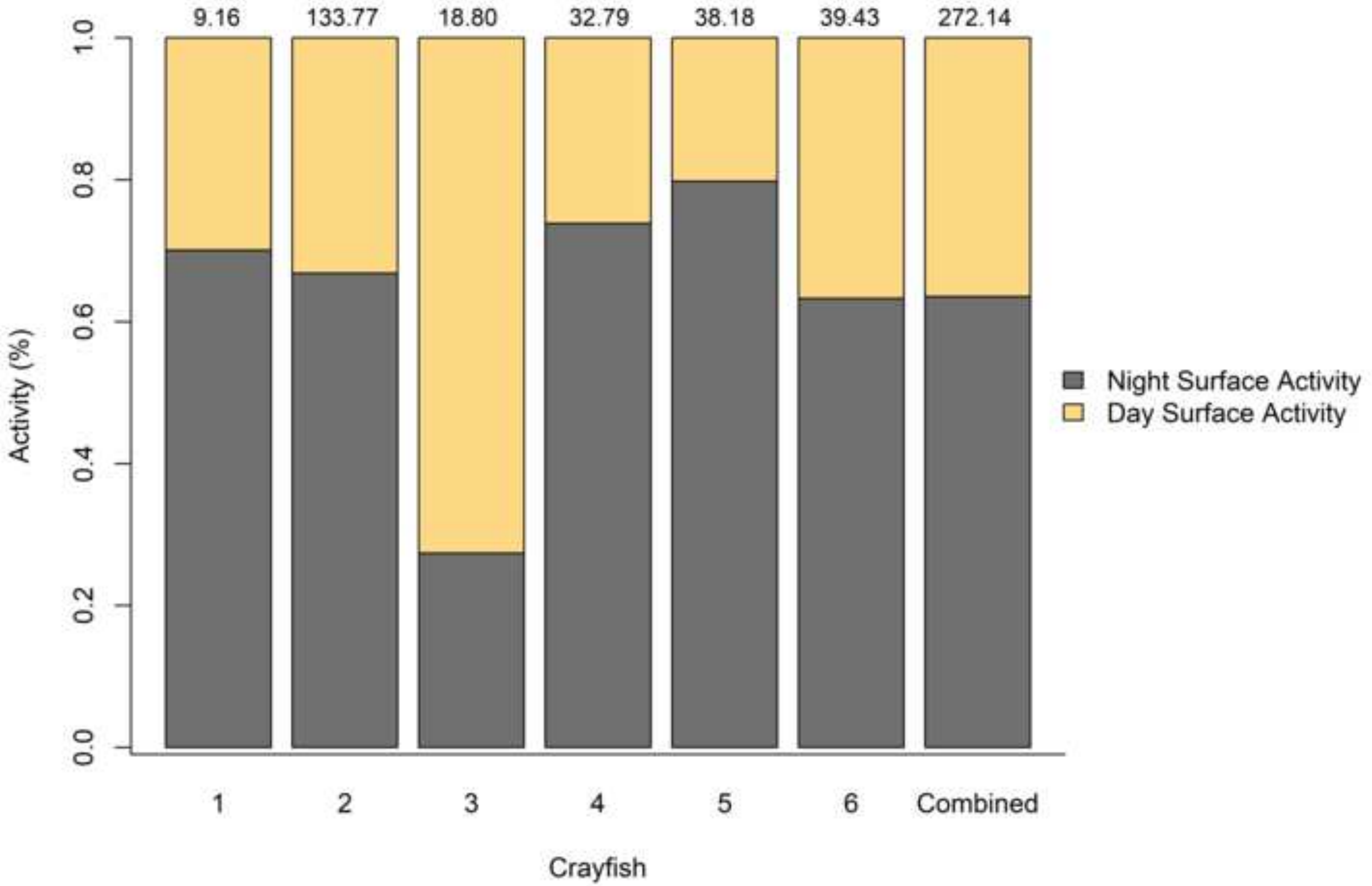

Figure 7

Click here to access/download;Figure;Fig7.tif

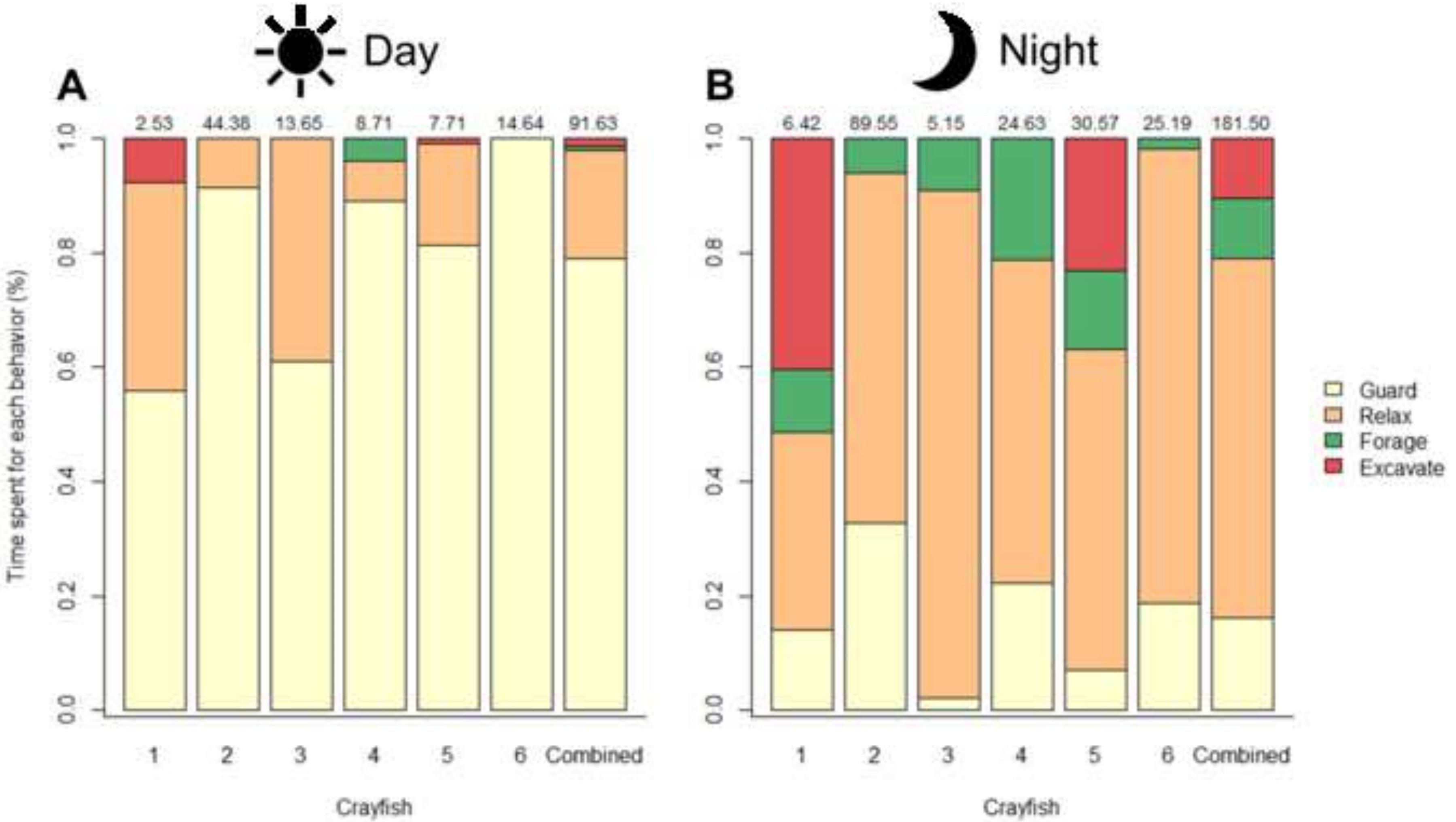

Figure 8

Click here to access/download;Figure;Fig8.tif

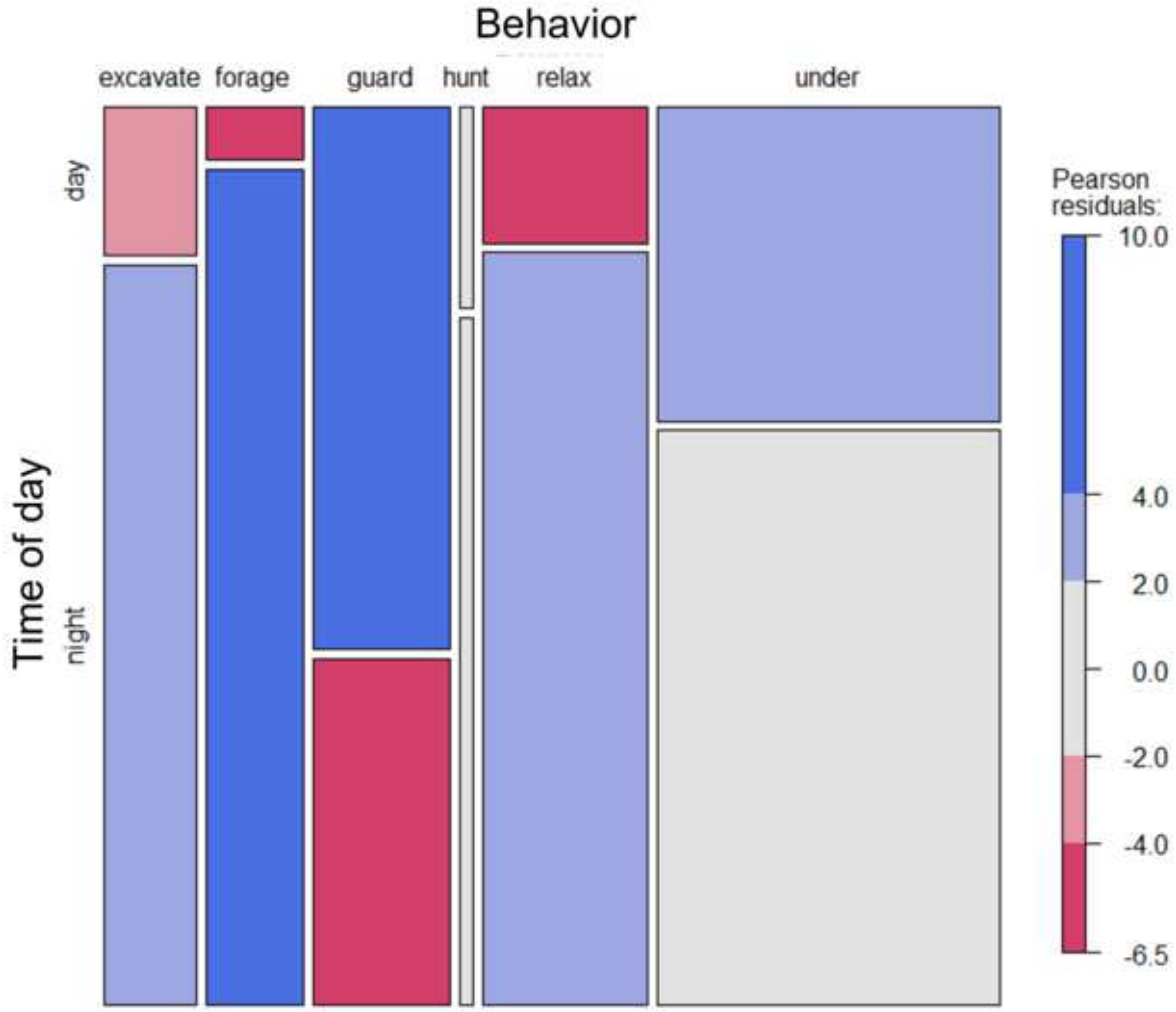

File S1

Click here to access/download
Supporting Information
File S1.mp4

File S2

Click here to access/download
Supporting Information
File S2.mp4

File S3

Click here to access/download
Supporting Information
File S3.mp4

Figure S1

Click here to access/download
Supporting Information
FigureS1.png

Figure S2

Click here to access/download
Supporting Information
FigureS2.png

Figure S3

Click here to access/download
Supporting Information
FigureS3.png

Figure S4

Click here to access/download
Supporting Information
FigureS4.png

On the surface or down below: Field observations reveal a high degree of surface activity in a burrowing crayfish, the Little Brown Mudbug (*Lacunicambarus thomai*)

Kaine M. Diehl [1], Nicoleena M. Storer [1], Hogan D. Wells [1], Destinee A. Davis[1], Zachary J. Loughman[1] & Zackary A. Graham[1]*

[1] Department of Organismal Biology, Ecology, & Zoo Science
West Liberty University, 208 University Dr. West Liberty, WV 26074
*Corresponding Author: Zackary.graham@westliberty.edu

Abstract

Opposed to most crayfish species that inhabit permanent bodies of water, a unique burrowing lifestyle has evolved several times throughout the crayfish phylogeny. Burrowing crayfish are considered to be semi-terrestrial, as they burrow to the groundwater—creating complex burrows that occasionally reach 3 m in depth. Because burrowing crayfishes spend most of their lives within their burrow, we lack a basic understanding of the behavior and natural history of these species. However, recent work suggests that burrowing crayfishes may exhibit a higher level of surface activity than previously thought. In the current study, we conducted a behavioral study of the Little Brown Mudbug, *Lacunicambarus thomai* using video surveillance to determine their degree of surface activity and behavioral patterns. Throughout 664 hrs of footage, we observed a surprisingly high amount of activity at the surface of their burrows—both during the day and night. The percentage of time that individual crayfish ~~were~~ was observed at the surface ranged from 20.55% to 68.75% per individual, with an average of 42.48% of the time spent at the surface across all crayfish. Additionally, we created an ethogram based on six observed behaviors and found that each behavior had a strong circadian effect. For example, we only observed a single observation of foraging on vegetation during the day, whereas 270 observations of this behavior were documented at night. Overall, our results suggest that burrowing crayfishes may exhibit higher levels of surface activity than previously thought. To increase our understanding of burrowing crayfish behaviors ecology, we encourage the continued use of video-recorded observations in the field and the laboratory.

Keywords: primary burrowing crayfish, behavioral ecology, behavior, crustacean, ethogram

Introduction

Crayfish are a diverse group of freshwater crustaceans with over 700 species globally (1,2). Many crayfishes are considered to be model organisms for behavioral biology due to their ease of capture, suitability for laboratory studies, simple neural architecture, and complex behavioral repertoires (3–7). However, this work stems from studies of a few model species from North America (i.e., *Procambarus clarkii*, *Procambarus virginalis*, *Faxonius virilis*); all of which inhabit lentic and lotic aquatic environments. Although most crayfish species inhabit surface water systems like streams, lakes, rivers, ponds, and marshes, crayfishes have repeatedly evolved a semi-terrestrial burrowing lifestyle throughout their phylogeny (8–10). Despite all species of crayfish possessing the ability to burrow to some degree (11), the complexity and reliance on these burrows vary greatly both within and between species (12,13).

Crayfishes have historically been assigned to three different ecological classifications based on their burrowing ability and their reliance on their burrows: tertiary burrowing species, secondary burrowing species, and primary burrowing species (12,14). Tertiary burrowing crayfishes are species which rely on permanent bodies of water and are only capable on constructing relatively simple burrows underneath benthic substrates. Secondary burrowing species can also inhabit permanent bodies of water, but during periods of drawdown they are capable of excavating complex burrows to access groundwater. Primary burrowing species (hereafter referred to as burrowing crayfish) are often considered semi-terrestrial as they are highly reliant on their burrows for their entire life; as they potentially serve as a location for breeding, refuge from predators, and as a location to acquire and cache food (12,13). Burrowing crayfish burrows are often extremely complex and may have several entrances (i.e., portals),

tunnels, and chambers that can reach several meters deep (12,13). In addition to providing shelter for the crayfish that construct them, such burrows can serve as necessary habitat for other organisms (15–18) and increase soil bioturbation and habitat complexity (19–21).

Compared to our knowledge of tertiary and secondary crayfishes that occur in lentic and lotic environments, our understanding of burrowing crayfish behavior is scant (13,22). Historically, burrowing crayfish were thought to spend nearly their entire lives below the surface, and thought to occasionally leave their burrows for brief terrestrial excursions to search for food or to find a mate (12). Minimal surface activity and their propensity to retreat to their burrow when startled has led to difficulties in capturing and observing burrowing species (23,24). Resultant of their reclusive lifestyle, our understanding of burrowing crayfish activity patterns and behavior is minimal, with much of the work on these species being derived from *in situ* natural history observations (12,25–28) and not systematic investigations. However, despite these difficulties, semi-naturalistic mesocosms and custom-built observation chambers have been useful in the study of burrowing crayfish behavior (29–33).

Burrowing crayfish are considered to be nocturnal (34), which further complicates our ability to adequately study their behavior. But in some instances, burrowing crayfish are observed at the surface of their burrow during the daytime (12,34–36). Interestingly, recent studies reveal that burrowing crayfish may exhibit a higher level of surface activity than previously thought (35,37). Bearden et al. (2020) conducted the most comprehensive study to date on the activity of burrowing crayfish (37). Using motion-trigger laser photography in two species of burrowing crayfish (*Lacunicambarus erythrodactylus* and *Procambarus holifieldi*), Bearden and colleagues measured activity throughout a one-year period. In their study, activity was considered based on whether the crayfish was visible at the entrance of their burrow (37).

This study confirmed that nocturnal activity occurred most frequently, specifically during times of cool groundwater temperature and warm air temperatures (37). Furthermore, despite observing substantial activity at night for both species, there were many occurrences of daytime surface activity throughout their study.

Bearden *et al*. (2020) provides robust evidence regarding the environmental correlates of activity in these two species and serves as a solid foundation to build off for future studies on burrowing crayfish surface activity. However, the use of photography and not videography limited their insights into crayfish surface behaviors. As such, the photographs made defining behaviors difficult, and limited behavior determination to chimney construction and surface activity (37). As stated previously, Bearden *et al*. (2020) goals were not specific to defining burrowing crayfish surface behaviors, but rather determining if surface activity was correlated to specific environmental correlates. Therefore, the behavioral complexity of burrowing crayfishes still remains unknown. Given their utilization of complex burrow systems and evidence of social behavior (13), burrowing crayfishes species may exhibit complex ritualistic behaviors like non-burrowing crayfishes (3,13,38). However, no study to date has conducted long-term behavioral observations of a burrowing species in a completely natural environment.

Here, we conducted a behavioral study of a burrowing crayfish species through video-recorded field observations. Specifically, we monitored the surface ~~of burrows~~of burrows inhabited by the Little Brown Mudbug, *Lacunicambarus thomai* (Fig 1). *Lacunicambarus thomai* is as a burrowing crayfish species with a high propensity to inhabit burrows in marshes, roadside ditches, and flooded fields (39). Populations of *L. thomai* often live in localized colonies with conspecifics and inhabit burrows that are relatively simple but can nonetheless be up to 1-1.5 m deep (39,40). Compared to most burrowing crayfish species, *L. thomai* and other members of the

genus *Lacunicambarus* seem to exhibit a high degree of surface activity (35,36,39–41), which makes them ideal for our investigations. Overall, we hope to shed light on the surface behavior of *L. thomai* by 1) determining the prevalence of surface activity and 2) creating an ethogram specific to burrowing crayfish, and 3) analyzing the behavioral repertoire and prevalence of behaviors exhibited by *L. thomai*.

Fig 1. Photographs of adult burrowing crayfish, The Little Brown Mudbug, *Lacunicambarus thomai,* exhibiting daylight activity outside of its burrow. The burrow's entrance portal is out of sight due to the extensive leaf litter coverage. Photographs courtesy of John Freudenstein.

Methods

Study site

In June and July 2020, we conducted non-stop 24-hour video surveys within a population of the Little Brown Mudbug, *Lacunicambarus thomai* (Fig 1) in Hickory, Pennsylvania, U.S.A. This population of *L. thomai* is located on the edge of a man-made pond on a private, residential yard. More specifically, our study site was located in a dense patch of grasses on the edge of the pond with no canopy cover. Within this location, the water table remains shallow throughout the year and there is an estimated population of 20 *L. thomai* within an area of ~20 m^2. Burrows were sporadically located throughout the population and were ~1 m to ~ 5 m from one another. No other crayfish species have been collected or identified from this location and no crayfish have ever been collected from the pond. We chose which burrows to record based on evidence of

Formatted: Font: Italic

Formatted: Font: Italic

Formatted: Superscript

recent surface activity (activity observed at burrow portal during preliminary night-time observation), as well as whether the burrow portal was visible from an overhead view. Because capturing burrowing crayfish often destroys the burrow and the population was relatively small, we chose to not capture the individuals ~~in this portion of our study~~. Therefore, we do not report morphological or demographic data from the recorded footage (i.e., sex, body size). However, based on the circumference of the burrow tunnels as well as the video footage, all burrows in which we recorded (see below) were from adult *L. thomai*. Importantly, although other burrowing crayfish species are known to exhibit social behavior and have multiple individuals within a single burrow, *Lacunicambarus* spp. seldomly exhibit social behavior and therefore it is assumed that each burrow only housed a single crayfish (41, but see 42).

Because our work was conducted on privately owned property on an unlisted crayfish species and we did not collect any specimens for this project, no specific permits were necessary for our research.

Videography

Overtop each burrow that we recorded, a custom-built video camera mount was constructed by placing a Reolink 5MP video camera inside of a 5-gallon bucket that we mounted with wire approximately 0.5 m above the burrow (Fig 2). These camera mounts protect the camera from the external environment and reduce glare. We recorded each crayfish until their burrow was either capped or until there was a video camera malfunction. If a crayfish burrow contained more than a one portal, we only mounted the video camera overtop the larger, primary portal which is where burrowing crayfish exhibit nighttime activity (*Z. Loughman*, personal communication). In

Formatted: Font: Italic

Formatted: Font: Italic

Field Code Changed

Formatted: Font: Italic

total, we recorded and reviewed (see below) 664.7 hr of footage from ~~6~~ six adult *L. thomai* (Table 1). The length of time recorded for each crayfish ranged from 44.5 6 hr to 263.82 hr (Table 1).

Fig 2. Images representing our custom video surveillance mount. (A) Diagram of the setup of our video surveillance setup used to monitor crayfish behavior. (B) Photograph of the setup *in situ* **C)** Photograph of the view of the camera mounted from inside of the bucket.

Table 1. Dates and total time that each crayfish was filmed throughout the duration of our study. Only a single crayfish (Crayfish 5) was filmed discontinuously due to failure of the video recorder.

Crayfish		
	Dates Filmed (MM/DD/YYYY)	Total Time Filmed (hr)
1	06/03/2020 – 07/02/2020	44.56
2	07/02/2020 – 7/20/2020	263.82
3	06/28/2020 – 06/30/2020	40.34
4	07/01/2020 – 07/06/2020	105.93
5	06/13/2020 – 06/14/2020;<br>06/28/2020 – 07/03/20	151.73

6	07/21/2020 – 07/24/2020	57.35
Combined	-	664.73

Ethogram

We reviewed all video footage with Windows Movie Editor Software. While reviewing the footage for each crayfish, we created an ethogram based on observed behaviors (File S1; File S2; File S3). In total, our ethogram contained 5 surface activity behaviors, as well as a single “inactive” behavior (under) which was used to denote when the crayfish was not visible at the surface of the burrow. See Table 2 for detailed descriptions of each behavior. Then, using our ethogram, we recorded what behavior each crayfish was exhibiting, the duration of the behavior, and the time in which the crayfish started and ended the behavior. We also noted whether each crayfish exhibited behaviors during the day or night based on the civil twilight time listed for Hickory, Pennsylvania, U.S.A (collected from www.time.unitarium.com). For our study, we considered a crayfish to be active if they were visible at the surface of their burrow (Fig 3; (35)). We considered a crayfish to be considered inactive if they were not visible at the surface of their burrow. We are unable report on any below-surface activity within our study.

Table 2. Ethogram used to quantify the behaviors from recordings of *Lacunicambarus thomai*.

Behavior	Description
Relax	Crayfish is motionless and partially out of burrow portal with both claws in a “U” orientation directed anteriorly. Movements consist of simple shifts in body position.

Guard	Crayfish is motionless and resting on burrow portal lip (more exposed during the night) with both claws in a "V" orientation. Both claw tips touching or nearly touching directly anterior to the rostrum.
Forage	Crayfish is actively manipulating and foraging for plant material (leaves, grass, or roots).
Excavate	Crayfish is actively carrying, moving, or placing burrow substrate outside of the burrow beyond the burrow portal.
Hunt	Crayfish quickly rushes beyond the burrow portal in attempt to predate on living animal prey.
Under	The crayfish is inactive on the surface and not visible.

Fig 3. Example of relax and guard behaviors from video surveys *of Lacunicambarus thomai*. The top two images (A) and (B) represent crayfish in the relax position, whereas the bottom two image (C) and (D) represent the crayfish in the guard position. Red circles and red lines are presented to give a representation of the differences in articulation between the relax and the guard pose. Photos (A) and (C) were taken at night, whereas photos (B) and (D) were taken during the day.

Environmental data

Because we wanted to investigate broad-scale trends of how the surface activity of *L. thomai* varied with environmental conditions, we gathered environmental data from an online source (www.weatherunderground.com). We collected data from the closest available weather station in

Imperial, Pennsylvania, U.S.A (~24 km away). Specifically, the environmental data that we collected from this source was air pressure, precipitation, temperature, and humidity. Later, these variables were used as covariates to determine which environmental variable best predicted *L. thomai* surface activity.

Statistical analysis

We conducted all statistical analyses in R version 3.5.1 (44). For all of the models described below, we visually inspected our data using the performance package in R to assess normality and collinearity of our model fits (45). For descriptive analyses of *L. thomai* behavior, we calculated the frequency, mean, and range of each behavior. Additionally, we wanted to describe the activity of *L. thomai* based on the time of day. Because of the uneven distribution of video footage length for each crayfish (Table 1), we decided to report the majority of our results in terms of percentages, and not raw values. Therefore, we calculated the ~~frequency~~ percentage of crayfish which exhibited surface activity at each hour by dividing the number of hourly observations in which surface activity was observed by the total number of hours which we recorded throughout our entire study. ~~in which crayfish were active during each hour throughout the entire duration of our study.~~ We averaged these values together for all crayfish which enabled us to investigate patterns in surface activity based on the hour of the day. The same procedure was conducted to determine the percentage of time that each crayfish was observed at the surface throughout the daytime and nighttime.

To determine which environmental variables best related to burrowing crayfish activity, we initially used a model selection procedure with several linear mixed effect models with

activity as dependent variable and the environmental variable (i.e., air pressure, precipitation, temperature, and humidity) as the independent variable. For this analysis, we determined a crayfish to be active during a given hour if they were recorded at the surface of their burrow within any point during that hour. In these models, we included crayfish ID as a random effect. Based on the Akaike information criterion (AICc) of these models, we found that only temperature and humidity effected activity of burrowing crayfish, and therefore we proceeded with these two variables for the remainder of this analysis.

Thus, to evaluate how environmental variables (i.e., humidity and temperature) and time of day (i.e., hour) influenced activity of *L. thomai*, we used multimodal averaging to determine how temperature, humidity, and time (independent variables) influenced the activity of crayfish (dependent variable). For this analysis, we determined a crayfish to be active during a given hour if they were recorded at the burrow portal within any point during the hour. Additionally, because there were multiple observations from each crayfish, we included crayfish ID as a random effect to control for individual differences in individual activity.

We first fit the full model to the data with the *lme4* library of the R statistical package (46). The full model contained temperature, humidity, time, and the interaction between these three variables as the independent variable. Because our dependent variable (i.e., activity) was coded as 1 (active) or 0 (inactive) we used a binomial distribution of error in this model. This model also included crayfish ID as a random variable. Then, we fit models that contained a subset of the terms in the full model. For each model, we calculated the corrected Akaike information criterion and the Akaike weight (i.e., the probability that the model described the data better than the other models in the set). Finally, we used the parameters of each model and Akaike weight to calculate a weighted average of each parameter. These model-averaged

parameters enable one to determine the expected probability for any combination of values for the fixed factors in the full model. This approach eliminates the use of *P* values because all models (including the null model) contributed to the expected probabilities. Importantly, collinearity was unlikely to bias our model averaged parameters, (47); because none of our independent variables (humidity, temperature, and time) were not strongly correlated (all r < 0.6).

To determine whether each specific behavior was more or less likely to occur during the day or night, we conducted ~~6~~ six separate Chi-square tests of independence. We conducted 5 separate chi-squared tests for each individual behavior (relax, guard, forage, excavate, hunt, and under). We also performed a single chi-squared test for all ~~6~~ six behaviors combined to determine whether all activity was more likely to occur during the day or the night. Additionally, we analyzed the likelihood of each behavior occurring during the day or night with a contingency mosaic plot (48). Continency mosaic plots display the Pearson residual values as colors in relation to the deviation between expected (equally as likely to occur during the day and night) versus the observed frequencies (48). This allows us to both visually and statistically investigate the directionality of the observed frequencies of each behavior (and the combined behaviors) in relation to equal frequencies.

Results

Hourly surface activity

~~Throughout our study, we~~ We observed *L. thomai* exhibiting surface activity throughout every single hour of the day. However, there was variation in the times that *L. thomai* were most likely

to be observed active at burrow portals. Surface activity peaked from 20:00 hr – 03:00 hr, with nearly every single crayfish active during those times during the study (Fig 4). After 03:00 hr, crayfish activity slowly declines with surface activity rarely being observed at 17:00hr. Despite being less active throughout the day, 25% to 50% of time each crayfish remained active throughout the daytime.

Fig 4. The percent of *Lacunicambarus thomai* (n=6) that were active for each hour during our study. Gray areas represent the civil twilight time for our study period that represents the transition between day and night. Crayfish were more likely to be active during the night than during the day. Data is reported from all crayfish throughout the entire period of our study.

Environmental predictors of surface activity

Lacunicambarus thomai surface activity was best predicted by a model containing the effect of time, humidity, temperature, an interaction between time and humidity, and an interaction between humidity and temperature (Table 3). However, the three most likely models were all ~~were~~ within 2 AICc values of the best fit model (Table 3). Based on the model averaged coefficients, humidity, temperature, and the interaction between humidity and temperature all predicted the activity of *L. thomai* throughout the duration of our study (Table 4). However, the humidity variable had the strongest overall effect (Table 4). Visualizations of the environmental variables are depicted in the Supplemental Informaton (Figure S1, Figure S2, Figures S3, Figure S4).

Table 3. The most likely model predicting the activity of burrowing crayfish based on time, humidity, temperature, an interaction between time and humidity, and an interaction

between humidity and temperature. For each model, degrees of freedom (d.f.), the corrected Akaike information criterion (AICc), Akaike weight (*w*), and the log likelihood (ll) are reported. Models were ranked according to their corrected Akaike information criterion (AICc). Besides the null model, only models with a *w* > 0.00 are reported.

model	d.f.	ll	AICc	DAICc	*w*
Time + humidity + temperature + time*humidity + humidity*temperature	8	-367.90	752.00	0.00	0.42
Time + humidity + temperature + humidity*temperature	7	-369.90	753.96	1.96	0.16
Time + humidity + temperature + time*humidity + time*temperature + humidity*temperature	9	-367.88	754.01	2.00	0.15
Time + humidity + temperature + time*humidity + time*temperature + humidity*temperature + time*humidity*temperature	10	-367.08	754.47	2.47	0.12

Humidity + temperature + humidity*temperature	6	-371.61	755.34	3.34	0.08
Time + humidity + temperature + time*temperature + humidity*temperature	8	-369.70	755.61	3.61	0.07
null	3	-482.07	970.17	218.16	0.00

Table 4. Coefficients and standard errors (SE) for the model predicting the activity of *L. thomai* based on full model averaging. Coefficients are reported in order based on their absolute values. Humidity was the strongest predictor of activity. Thus, the activity of *L. thomai* was negatively related to the ~~activity ~~degree of environmental humidity~~of crayfish~~.

Independent variable	Coefficient	SE
Humidity	-2.908	1.36
Humidity*temperature	0.057	0.019
Temperature	-0.045	0.012
Time*humidity	0.005	0.089
Time	0.003	0.053
Time*humidity*temperature	< -0.001	0.001

Time*temperature	< 0.001	0.001

Surface activity

Among the ~~6~~ six crayfish observed, there was a high degree of surface level activity. Each crayfish spent at least 20% of the time that we filmed at their burrow portal. The percentage of total time each crayfish was observed at the surface ranged from 20.55% (9.18 hr) of the total filmed time to 68.75% (39.42 hr); with an average of 42.48% of the time spent active across all ~~6~~ six crayfish (Fig 5). Interestingly, one of our ~~6~~ six crayfish (crayfish 6) spent a greater portion of the time active above ground than underground. The remaining 5 *L. thomai* spent a greater percentage of time underground, with a range of 31.25% (17.92 hr) to 79.44% (120.47 hr) of their time spent underground and with an average of 57.52% of each crayfish's time spent underground (Fig 5).

Formatted: Font: Not Italic

Fig 5. The percentage of time that *Lacunicambarus thomai* spent ~~time~~ visible at the surface of their burrow (Active at Surface~~active~~) or not visible underground in their burrow (~~inactive~~Underground). We report the percentage of time that each crayfish individual spent active or inactive as well as a combined total for all ~~6~~ six crayfish. For each crayfish, the total number of time (hours) that each crayfish was filmed is listed above each bar.

Day vs. night surface activity

All *L. thomai* exhibited a high degree of surface activity both during the day and during the night. The percentage of time that crayfish were active during the daytime ranged from 20.20%

(7.71 hr) to 72.61% (13.65 hr) of the time with an average of 36.46% of activity occurring at the burrow portal or on the surface (Fig 6). Regarding nighttime activity, five ~~5~~ of the ~~6~~ six crayfish exhibited more nighttime activity at the surface of their burrow than they did daytime activity. Night activity ranged from 27.38% (5.15 hr) to 79.79% (30.46 hr) with an average of 63.35% of the total surface activity occurring during the night (Fig 6).

Fig 6. The percentage of time that *Lacunicambarus thomai* was observed exhibiting surface activity~~e~~ during the day and during the night. We report the percentage of time that each individual crayfish spent during the day and the night as a combined total for all ~~6~~ six crayfish. For each crayfish, the total number of time (hours) that each crayfish was filmed active is listed above the bar.

The behaviors exhibited by *L. thomai* varied depending on whether they were active during the day or night (Table 5). The most common diurnal behavior recorded was the guard and relax behavior, which comprised nearly 80% of the total time crayfish were observed at the surface during the day (Fig 7). Interestingly, a single crayfish (crayfish 4) exhibited foraging behavior during the day for 3.9% (0.34 hr) of the time that they were filmed. We observed two crayfish (crayfish 1 and 5) excavating their burrow during the day. Overall, each crayfish spent most of their day activity exhibiting the guard behavior at their burrow portal (Fig 3; Table 5).

Table 5. The number of observations and the mean duration~~ing~~ of each behavior observed during our study. Each behavior is split based on whether the behavior occurred during the day or night. Data is reported based on the mean duration of each behavior from all six crayfish in our study. ~~Because of the discrepancy in the mean duration of each behavior, the mean durations of relax, guard, forage, and under are reported in hours, whereas the mean duration of hunt and excavation are reported in seconds.~~

Behavior	Day		Night	
	No. observations	Mean duration	No. observations	Mean duration
Relax	63	648 s ~~0.18 hr~~	320	~~0.31 hr~~1116 s
Guard	183	9468 s ~~2.63 hr~~	134	~~0.22 hr~~792 s
Forage	1	360 s ~~0.10 hr~~	227	~~0.12 hr~~432 s
Excavation	10	102.4 s	199	166.39 s
Hunt	3	120.33 s	29	80.72 s
Under	260	4572 s~~1.27 hr~~	509	~~0.14 hr~~504 s

Fig 7. The percentage of time that each *Lacunicambarus thomai* spent exhibiting different behaviors throughout the (A) day and the (B) night. We report the percentage of time that each individual crayfish spent during the day and the night and combined ~~6~~ six crayfish. The hunt behavior was not included in because of the relatively small time that this behavior was exhibited. For each crayfish, the total number of time (hours) that each crayfish was filmed active is listed above the bar.

Crayfish showed a greater level of nocturnal surface activity. The majority of their time was spent in the relax position during the night, and guarding was more likely to occur during the night compared to during the day (Fig 7); both of these behaviors occurred at burrow portals. Interestingly, each crayfish spent a portion of their nighttime activity exhibiting foraging behavior, with an average of 10.5% of all night activity including foraging (Fig 7). The same two crayfish that excavated during the day (crayfish 1 and crayfish 5) were also observed excavating their burrow at night, but they excavated for a greater percentage of their nighttime activity than their daytime activity (Fig 7).

When analyzing the likelihood of each behavior occurring at day versus night, the chi-squared analyses demonstrated that every ~~single~~ behavior was more likely to occur during one light period compared to another. Specifically, the guard behavior and inactivity (i.e., under) were more likely to occur during the day compared to the night (Fig 8; Table 6). By contrast, excavate, forage, and the relax behavior were more likely to occur at night (Fig 8; Table 6). The hunt behavior was also more likely to occur during the night than during the day (Fig 8; Table 6).

Fig 8. Mosaic plot of the behavior exhibited by each crayfish (excavate, forage, guard, hunt, relax, under) based on the time of day it was recorded (night, day). The width of the bars represents the number of observations that we recorded, whereas the height of the bars represents the number of observations that were recorded in the night (the bottom bar) versus the day (the top bar). The color of each bar represents whether the observed frequency of each behavior deviates from the expected frequencies if the variables were independent. Red denotes observed frequencies that are smaller than the expected frequency, whereas blue represents the observed frequencies that are larger than the expected frequencies. Gray represents a similar observed frequency when compared to the expected frequency.

Table 6. Results from Chi-squared test for the number of behaviors that occur during the day and versus the night. For each behavior, we report the degrees of freedom (d.f.), Chi-squared value ($\chi2$), and *p* value. All behaviors showed significant differences between the number of times the behavior was observed during the day or night.

Behavior			
	d.f.	$\chi2$	*p*
Relaxed	1	178.94	0.002
Guard	1	15.01	< 0.001
Forage	1	171.07	< 0.001
Excavate	1	92.45	< 0.001
Hunt	1	9.32	< 0.001
Under	1	66.16	< 0.001
All behaviors	5	269.29	< 0.001

Discussion

Out of the 664.73 hrs that we observed across six *L. thomai*; crayfish were active at the surface of their burrow nearly half of this time. Furthermore, we observed a surprisingly high amount of surface activity during the day, with nearly one-third of all surface activity occurring during the day and not at night. With our footage, we were also able to create an ethogram based on the ~~fve~~five different surface behaviors (relaxed, guard, forage, excavate, and hunt) that we observed.~~observed surface behaviors.~~ Our ethogram allowed us to examine the behavioral

repertoire and prevalence of the whether the observed behaviors ~~were~~ more frequently occurred during the day or night. We discuss these findings in relation to previously published observations of burrowing crayfish surface behavior as well as potential opportunities for future research.

Before our study, little has been reported on the degree of surface activity and specific behaviors exhibited by burrowing crayfishes. Although surface activity has been observed throughout burrowing crayfishes both during the day and during the night, the prevalence in which *L. thomai* was active at the surface far exceeds that of other previously published reports. Previous accounts suggest that burrowing crayfish were only active above ground during a few instances which included high water events (50), during cloudy days according to light levels (35), or when looking for food or mates (12). However, many of these accounts come from *in situ* natural history observations, and not systematic investigations. For example, Loughman *et al*. ~~2018~~ 2015 reports over 50 observations of *Cambarus pauleyi* at the surface of their burrow, but only three of these observations were made during the day (51). Moreover, Foltz *et al*. 2018 states that *Cambarus loughmani* did not rise from burrows until a few hours after twilight, with no daylight activity reported (26).

Although we studied a small number of *L. thomai* ~~behavior~~ during a two-month summer period, every ~~single~~ crayfish was active during peak activity times. Only two previous studies have reported hourly activity data for burrowing crayfishes (34,37). For over a year, Bearden *et al*. (2021) collected hourly activity data for two burrowing crayfish species (*Lacunicambarus erythrodactylus* and *Procambarus holifieldi)*. In their study, both *L. erythrodactylus* and *P. holifieldi* were observed active roughly 25% of the time during the night and were rarely observed being active during daytime hours (37). In a laboratory study, Palaoro *et al*. 2013

collected hourly data on a South American burrowing species (*Parastacus brasiliensis*) and showed that this species spent a majority of their experiment within their burrow, but were more likely to leave their burrow during the night compared to during the day (34).

In our study, the high degree of surface activity observed may be specific to crayfish in the genus *Lacunicambarus*. However, because our study was conducted on few individuals, future research on the surface behavior of *Lacunicambarus thomai* and the other 13 species in the genus *Lacunicambarus* is warranted (52). Alternatively, other burrowing species (and genera) may also be highly active during the day, but researchers often exclusively collect burrowing crayfish during the night which can bias the prevalence of these behavioral observations (24,53). For example, in a seven year-long ecological study of the Digger Crayfish, *Creaserinus fodiens*, Norrocky *et al*. 1991 reported that all crayfish collections occurred at night (54). Clearly, the high degree of surface activity that we found in *L. thomai* contrasts our previous understanding of the activity and surface behaviors of burrowing crayfishes. Further, our study solely reports observations of surface behavior, and we are unable to report on the presence, absence, or degree of underground activity.

Based on our analysis of the environmental predictors of surface activity, we found that humidity had the strongest overall effect of predicting burrowing crayfish surface activity. Specifically, we found that crayfish were more likely to be active at lower humidity values. These results contrast previous findings by Bearden et al. 2021, in which activity was best predicted by a combination of variables, including daylength, air temperature, and groundwater temperature. Importantly, our analysis only uses reported data from the macro-environment at a nearby weather station, and not local, micro-environmental variables (variables recorded at the exact environment of or study site), as was done by Bearden and colleagues (37). For example,

Formatted: Font: Italic

Formatted: Font: Italic

Formatted: Indent: First line: 0"

in their study, Bearden et al. (2021) found that chimney constructed was related to precipitation events. It remains unknown whether the other surface behaviors which we describe have similar correlations to environmental variables such as prescription or groundwater temperature. Future studies should take into account the variation within environmental variables such as air temperature and burrow temperature to investigate behavioral patterns in burrowing crayfish. For example, activity in burrowing mammals is often best predicted by an interaction between both surface and groundwater temperatures (55). Therefore, similar surface and groundwater temperature interactions may occur in burrowing crayfishes which are already known to use their burrows as refuges from harsh environmental temperatures (12,13,37).

Besides our study, only one other study has created an ethogram specific to burrowing crayfish surface behavior, although this study was conducted in a laboratory environment (34). By contrast, our *in-situ* recordings allowed us to create an ethogram based entirely on natural behaviors, which revealed the ~~intricacies~~ richness of burrowing crayfish surface behavior—many of which have been previously undocumented. In our study, because *L. thomai* were constantly being monitored, we were able to categorize each crayfish into one of five different surface behaviors. The two most prevalent activities were the relax behavior and the guard behavior, which we separated based on the orientation of the crayfish’s claws relative to their body position (Fig 3). Whether the guard behavior’s function is truly to defend the burrow is unknown, because we did not observe any intraspecific interactions in our study. However, defense, guarding-like behavior has been reported in other burrowing crayfish (51).

Interestingly, there were strong differences in the prevalence of the guard and relax behaviors depending on whether it was during the day or night. We found that during the day, *L. thomai* was more likely to be active in the guarded position compared to the relax position. By

contrast, when we observed *L. thomai* active during the night, they were much more likely to be in the relax position than the guard position. The function of these different postural behaviors is unknown and requires further investigation. But we can speculate that the guard behavior is more likely to be exhibited during the day due to the increased potential for predator interactions. Indeed, we observed terrestrial predators (i.e., snakes and birds) interacting with *L. thomai* burrows during the day (36)~~(Z. Graham *et al*., in review)~~. Alternatively, the rest behavior being more likely to occur during the night may relate to this posture's increased ability to quickly capture live prey that move near the burrow (35,36,41) ~~(31; Z. Graham *et al*., in review)~~. Future studies must investigate whether or not these postural differences are present throughout other burrowing crayfishes, or if they are specific to *L. thomai*.

We also observed 271 instances of *L. thomai* engaging in active foraging for nearby vegetation. Within our observations, *L. thomai* often explored the area within the vicinity of their burrow portal and used their claws to cut nearby vegetation. Across all our observations, only a single instance of vegetation foraging occurred during the daytime, which demonstrates a strong preference for this behavior occurring at night when there are presumably fewer active predators. Interestingly, the vegetation was either immediately consumed at the base of the burrow or was taken down into the burrow out of the view of the camera. Therefore, our observations confirmed previous reports of burrowing crayfish foraging; because the area surrounding the burrow portals are often replete with vegetation (26). Furthermore, crayfish burrows are commonly excavated into large chambers, and large amounts of vegetation are found within the burrow tunnels and the burrow chambers, which are likely to serve as a food storage cache (26,27,56). Although our observations confirm that *L. thomai* does take vegetation down into the burrow it is difficult to confirm the hypothesis that *L. thomai* burrows may serve as a food cache. The use of crayfish

burrowing chambers in laboratory settings (29) ~~are~~ is required to confirm the possibility for crayfish using their burrows to store and consume food resources.

In addition to observations of *L. thomai* foraging for vegetation, we observed 32 instances of the crayfish exhibiting sit and wait hunting behavior. In these observations, the crayfish sat at the burrow portal and quickly lunged at live animal prey (i.e., the hunt behavior). In some instances, this ambush behavior successfully resulted in the capture of live animal prey, whereas other attempts did not result in successful acquisition of the prey. When this behavior resulted in a successful attempt, the crayfish immediately took the item into their burrow. This ambush behavior was more likely to occur nocturnally than diurnally, with only three of 32 observations occurring during the day. Interestingly, this sit and wait predatory behavior seems to be prevalent throughout the genus *Lacunicambarus*, as it has been reported in at least 2 other *Lacunicambarus* species (35,36,41) ~~(31; Z. Graham *et al*., in review)~~. Recently, Thoma (2022) reports that in Ohio, *Lacunicambarus chimera* is never active at the surface of their borrow, whereas another species, *Lacunicambarus nebrascensis* rarely exhibits surface activity (57). Although other species of crayfish have been observed capturing live animal prey (28,58), the degree and reliance on this ambush predation behavior across burrowing crayfishes is unknown.

Formatted: Font: Italic

Formatted: Font: Italic

Across the six crayfish that we studied, we only observed two *L. thomai* actively excavating their burrows. These observations indicate burrow excavation is not an exceedingly common *L. thomai* behavior (34,37), and may only occur when necessary alterations to the burrow architecture are needed. Each observation of excavation behavior always occurred in a repeated series of ritualized behaviors. First, crayfish would be observed bringing mud up to the burrow portal. Once at the surface, the crayfish would immediately stop before reaching ground level and immediately tap their antennae at their edge of their burrow portal. Lastly, the crayfish

would finally emerge and place the mud pellet and methodically use <u>its</u>~~tis~~ claws the push the mud into position.

In previous studies of *P. holifieldi*, precipitation was one of the variables that influenced the likelihood of burrow excavation (i.e., referred to as chimney construction in the study) (37). Indeed, burrows become flooded during times of rainfall and often require renovations following these events (Z. Loughman, personal observations). Only two small rain events occurred during our study period, which may explain the lack of burrow excavation being observed. Furthermore, excavation behavior was more likely to occur at night than during the day. Intuitively, burrow excavation is a relatively costly behavior, given substrates must repeatedly be excavated from the bottom of the burrow to the surface. Such behaviors are more likely to have increased costs during the day, both based on the high surface temperatures and the potential for increased rates of predation during the daytime.

In summary,<u> based on our preliminary investigation, we found that</u> *L. thomai* exhibits a high degree of surface activity~~ compared to other studied burrowing crayfish~~. Further, we found that *L. thomai* exhibits several behaviors at the their burrow portal, and these behaviors all were more likely to occur during either day or night. However, our study brings several unanswered questions to light regarding the behavior of burrowing crayfishes. For example, although we observed many small foraging excursions that occurred within the immediate vicinity of the burrow, we did not observe a single large-scale excursion during this time. In other species of burrowing crayfishes, substantial surface activity occurs immediately after rainfall, and such crayfish may travel large distances in a short period of time (12; Z. Loughman & Z. Graham personal observations). Although the exact function of these excursions is unknown, it is unclear whether or not *L. thomai* engages in such behaviors.

Further, throughout our study, we did not observe any intra-specific interactions. Again, scattered observations highlight the social behavior of burrowing crayfishes, but interestingly, we did not observe any social behavior throughout the course of our study. Future studies should conduct similar behavioral observations throughout the year and also investigate potential demographic differences in the behaviors we report. By expanding the time frame in which records occur, social interactions of larger foraging excursions may be recorded. Lastly, although our study has shed light on the activity of crayfishes at the surface, much remains unknown about the subsurface behavior of burrowing crayfishes. Additionally, since we discovered that this species displayed easily identifiable postural behaviors, this may allow for automated video analysis of body position, activity, and behaviors. As such, the continued use of burrowing crayfish observations chambers (29) will be paramount in furthering our understanding of the behavioral ecology of burrowing crayfishes. In conclusion, our study revealed a surprisingly high degree of surface activity in a species of burrowing crayfish. Based on the historical difficulties in studying burrowing crayfish, there is much to learn about the ecological and evolutionary dynamics of burrowing crayfish behavior.

Acknowledgements

We thank the Davis family for the use of their land for the study location.

Author Contributions

Conceived and designed the study: Z.J.L, D.A.D, K.M.D, Z.A.G. Collected the data: K.M.D D.A.D, H.D.W. Analyzed the data: K.M.D, Z.A.G. All authors contributed to writing the paper.

References

1. Richman NI, Böhm M, Adams SB, Alvarez F, Bergey EA, Bunn JJS, et al. Multiple drivers of decline in the global status of freshwater crayfish (Decapoda: Astacidea). Philos Trans R Soc B Biol Sci. 2015;370(1662):1–11.

2. Crandall KA, DeGrave S. An updated classification of the freshwater crayfishes (Decapoda: Astacidea) of the world, with a complete species list. J Crustac Biol. 2017;37(5):615–53.

3. Moore P. Agonistic behavior in freshwater crayfish: the influence of intrinsic and extrinsic factors on aggressive behavior and dominance. Evol Ecol Soc Sex Syst Crustac as Model Org. 2007;90–114.

4. Jimenez S, Faulkes Z. Establishment and care of a laboratory colony of parthenogenetic

Marbled Crayfish, Marmorkrebs. Invertebr Rearing [Internet]. 2010;1(1):10–8. Available from: http://inverts.info/content/establishment-and-care-laboratory-colony-parthenogenetic-marbled-crayfish-marmorkrebs

5. Seitz R, Vilpoux K, Hopp U, Harzsch S, Maier G. Ontogeny of the Marmorkrebs (marbled crayfish): A parthenogenetic crayfish with unknown origin and phylogenetic position. J Exp Zool Part A Comp Exp Biol. 2005;303(5):393–405.

6. Reynolds J, Souty-Grosset C. Management of freshwater biodiversity: Crayfish as bioindicators. Management of Freshwater Biodiversity: Crayfish as Bioindicators. 2011. 1–388 p.

7. Herberholz J, Antonsen BL, Edwards DH. A lateral excitatory network in the escape circuit of crayfish. J Neurosci. 2002;22(20):9078–85.

8. Stern DB, Breinholt J, Pedraza-Lara C, López-Mejía M, Owen CL, Bracken-Grissom H, et al. Phylogenetic evidence from freshwater crayfishes that cave adaptation is not an evolutionary dead-end. Evolution (N Y). 2017;71(10):2522–32.

9. Breinholt JW, Porter ML, Crandall KA. Testing Phylogenetic Hypotheses of the Subgenera of the Freshwater Crayfish Genus Cambarus (Decapoda: Cambaridae). PLoS One [Internet]. 2012 [cited 2019 Feb 5];e46105. Available from: www.plosone.org

10. Moore MJ, DiStefano RJ, Larson ER. An assessment of life-history studies for USA and Canadian crayfishes: identifying biases and knowledge gaps to improve conservation and management. Freshw Sci [Internet]. 2013;32(4):1276–87. Available from: http://www.journals.uchicago.edu/doi/10.1899/12-158.1

11. Berrill M, Chenoweth B. The Burrowing Ability of Nonburrowing Crayfish. Am Midl Nat. 1982;108(1):199–201.

12. Hobbs HH. The Crayfishes of Georgia. Smithson Contrib to Zool. 1981;(318):1–549.

13. Richardson AMM. Behvioral ecology of semterrestrial crayfish. In: Evolutionary ecology of social and sexual systems: Crustacae of model organisms. 2007. p. 219–338.

14. Hobbs HH. The crayfishes of Florida. Univ Florida Publicaiton, Biol Sci Ser. 1942;3(2):1–179.

15. Pintor LM, Soluk DA. Evaluating the non-consumptive, positive effects of a predator in the persistence of an endangered species. Biol Conserv. 2006;130(4):584–91.

16. Holycross AT, Mackessy SP. Variation in the Diet of Sistrurus catenatus (Massasauga), with Emphasis on Sistrurus catenatus edwardsii (Desert Massasauga) Author (s): Andrew T . Holycross and Stephen P . Mackessy Published by : Society for the Study of Amphibians and Reptiles S. J Herpetol. 2002;36(3):454–64.

17. Krupa JJ, Hopper KR, Nguyen MA. Dependence of the dwarf sundew (Drosera brevifolia) on burrowing crayfish disturbance. Plant Ecol [Internet]. 2021;222(4):459–67. Available from: https://doi.org/10.1007/s11258-021-01119-3

18. Creaser EP. Some cohabitants of burrowing crayfish. Ecology. 1931;12(1):243–4.

19. Welch SM, Waldron JL, Eversole AG, Simoes JC. Seasonal variation and ecological effects of camp shelby burrowing crayfish (Fallicambarus gordoni) burrows. Am Midl Nat. 2008;159(2):378–84.

20. Reynolds J, Souty-Grosset C, Richardson A. Ecological roles of crayfish in freshwater and

terrestrial habitats. Freshw Crayfish. 2013;19(2):197–218.

21. Richardson AMM. The effect of the burrows of a crayfish on the respiration of the surrounding soil. Soil Biol Biochem. 1983;15(3):239–42.

22. Graham ZA, Angilletta MJ. Distinguishing between unreliability and dishonesty: A comparitive study of aggressive communciation in crayfish. Funct Ecol. 2021;

23. Welch SM, Eversole AG. Comparison of Two Burrowing Crayfish Trapping Methods. Southeast Nat. 2016;5(1):27–30.

24. Ridge J, Simon TP, Karns D, Robb J, Fish US, Service W, et al. Comparison of three burrowing cray- fish capture methods based on relationships with species morphology, seasonality, and habitat quality. J Crustac Biol. 2008;28(3):466–72.

25. Glon MG, Thoma RF. An observation of the use of devil crayfish (Cambarus cf. diogenes) burrows as brooding habitat by Eastern cicada killer wasps (Sphecius speciosus). Freshw Crayfish. 2017;23(1):55–7.

26. Foltz DA, Sadecky NM, Myers GA, Fetzner JW, Welsh SA, Stocker GW, et al. Cambarus loughmani, a new species of crayfish (Decapoda: Cambaridae) endemic to the pre-glacial Teays River Valley in West Virginia, USA. J Nat Hist [Internet]. 2018;52(45–46):2875–97. Available from: https://doi.org/10.1080/00222933.2018.1557271

27. Loughman ZJ, Welsh SA, Thoma RF. Cambarus fetzneri sp. nov., a new species of burrowing crayfish (Decapoda: Cambaridae) from the Allegheny Mountains of Virginia and West Virginia, USA. Zootaxa. 2019;4651(1):38–50.

28. Loughman ZJ. Ecology of Cambarus dubius (Upland Burrowing Crayfish) in North-

central West Virginia. Southeast Nat. 2010;9(SPECIAL ISSUE 3):217–30.

29. Stoeckel JA, Helms BS, Cash E. Evaluation of a crayfish burrowing chamber design with simulated groundwater flow. J Crustac Biol. 2011;31(1):50–8.

30. Clay M, Stoeckel J, Helms B. The role of abiotic and biotic cues in burrow habitat selection by juvenile crayfish. Behaviour. 2017;154(12):1177–96.

31. Dalosto MM, Palaoro A V, Costa JR, Santos S. Aggressiveness and life underground : the case of burrowing crayfish. Behavior. 2013;150:3–22.

32. Costa JR, Dalosto MM, Palaoro A V, Santos S. Contest Duration and Dynamics are Affected by Body Size in a Potentially Subsocial Crayfish (Crustacea : Decapoda). 2016;122:502–12.

33. Grow L. Burrowing behaviour in the crayfish, Cambarus diogenes diogenes girard. Anim Behav. 1981;29(2):351–6.

34. Palaoro A V., Dalosto MM, Coutinho C, Santos S. Assessing the importance of burrows through behavioral observations of parastacus brasiliensis, a neotropical burrowing crayfish (crustacea), in laboratory conditions. Zool Stud. 2013;52(1):1–8.

35. Thoma RF, Armitage BJ. Burrowing Crayfish of Indiana. 2008;1–226.

36. Graham ZA, Diehl KM, Davis D, Loughman ZJ. Death from below : Sit-and-wait predatory behavior in a burrowing crayfish (Lacunicambarus thomai). Food Webs [Internet]. 2022;31(January):e00225. Available from: https://doi.org/10.1016/j.fooweb.2022.e00225

37. Bearden RA, Tompkins EM, Huryn AD, Schuster P. Motion-triggered laser photography

reveals fine- - scale activity patterns for burrowing crayfish. 2021;(January):1–13.

38. Florey CL, Moore PA. Analysis and description of burrow structure in four species of freshwater crayfishes (Decapoda: Astacoidea: Cambaridae) using photogrammetry to recreate casts as 3D models. J Crustac Biol. 2019;39(6):711–9.

39. Loughman ZJ, Simon TP. Zoogeography, taxonomy, and conservation of West Virginia's Ohio River floodplain crayfishes (Decapoda, Cambaridae). Zookeys. 2011;74(SPEC. ISSUE):1–78.

40. Loughman ZJ. Crayfishes of western Maryland: Conservation and natural history. Southeast Nat. 2010;9(SPECIAL ISSUE 3):33–62.

41. Simon TP, Morris CC. Cambarus (Lacunicambarus) erythrodactylus, a new species of crayfish (Decapoda: Cambaridae) of the Cambarus diogenes complex from Alabama and Mississippi, U.S.A. Proc Biol Soc Washingt. 2015;127(4):572–84.

42. Glon MG, Adams SB, Loughman ZJ, Myers GA, Taylor CA, Schuster GA. Two new species of burrowing crayfish in the genus Lacunicambarus (Decapoda: Cambaridae) from Alabama and Mississippi. Vol. 4802, Zootaxa. 2020. 401–439 p.

43. Miller JM, Niraula BB, Reátegui-Zirena EG, Stewart PM. Life history and physical observations of primary burrowing crayfish (Decapoda: Cambaridae) Cambarus (Lacunicambarus) diogenes and Cambarus (Tubericambarus) polychromatus. J Crustac Biol. 2014;34(1):15–24.

44. R Core Team: A Lang Environ Stat Comput. 2019;(R Foundat.

45. Lüdecke D, Ben-Shachar MS, Patil I, Waggoner P, Makowski D. performance: An R

package for assessment, comparison and testing of statistical models. J Open Source Softwater. 2021;6.60.

46. Bates DM. lme4: Mixed-effects modeling with R. 2010;

47. Cade BS. Model Averaging and Muddled Multimodel Inferences. Ecology. 2015;96(9):2370–82.

48. Meyer D, Zeileis A, Hornik K. The strucplot framework: Visualizing multi-way contingency tables with vcd. J Stat Softw. 2006;17(3):1–48.

49. Burnham KP, Anderson DR. Model Selection and Multimodel Inference: A Practical Information-Theoretic Approach. Springer; 2002.

50. Norrocky JM. Observations on the Ecology , Reproduction and Growth of the Burrowing Crayfish Fallicambarus (Creaserinus) fodiens (Decapoda : Cambaridae) in North-central Ohio. Am Midl Nat. 1991;125(1):75–86.

51. Loughman ZJ, Thoma RF, Fetzner JW, Stocker GW. Cambarus (Jugicambarus) pauleyi, a new species of crayfish (Decapoda: Cambaridae) endemic to southcentral West Virginia, USA, with a re-description of Cambarus (J.) dubius. Zootaxa. 2015;3980(4):526–46.

52. Hobbs L, Astacidea D, Glon MG, Broe MB, Crandall KA, Daly M, et al. Anchored hybrid enrichment resolves the phylogeny of Lacunicambarus Hobbs, 1969 (Decapoda: Astacidea: Cambaridae). 2022;42(January):1–18.

53. Loughman ZJ, Foltz DA, Welsh SA. Baited lines: An active nondestructive collection method for burrowing crayfish. Southeast Nat. 2013;12(4):809–15.

54. Norrocky MJ. Observations on the Ecology, Reproduction and Growth of the Burrowing

Crayfish Fallicambarus (Creaserinus) fodiens (Decapoda: Cambaridae) in North-central Ohio. Am Midl Nat. 1991;125(1):75.

55. Jannetti MG, Buck CL, Valentinuzzi VS, Oda GA. Day and night in the subterranean: Measuring daily activity patterns of subterranean rodents (Ctenomys aff. knighti) using bio-logging. Conserv Physiol. 2019;7(1):1–13.

56. Jezerinac RF, Stocker GW, Tarter DC. Crayfishes (Decapoda: Cambaridae) of West Virginia. Ohio Biological Survey,College of Biological Sciences, the Ohio State University; 1995.

57. Thoma RF. A Naturalist's Guide to the Crayfish of Ohio. Ohio Biological Survey; 2022.

58. Mccormack RB, Coughran J. Taxonomy, distribution and ecology of the Setose Yabby, Cherax setosus (Riek, 1951). Crustac Res. 2011;40(January):1–11.

Supporting information

S1 File. Recording of an excavation observation during the night.

S2 File. Recording of a foraging observation during the night.

S3 File. Recording of a hunt observation during the night.

S1 Figure. Average, Minimum, and Maximum Air Temperature at our study location in Hickory, PA throughout our study period (June 2020 – July 2020).

Formatted: Font: 12 pt

Formatted: Font: (Default) Times New Roman, 12 pt

Formatted: Line spacing: Double

S2 Figure. Average, Minimum, and Maximum Humidity at our study location in Hickory, PA throughout our study period (June 2020 – July 2020).

S3 Figure. Average, Minimum, and Maximum Air Pressure at our study location in Hickory, PA throughout our study period (June 2020 – July 2020).

S4 Figure. Precipitation at our study location in Hickory, PA throughout our study period (June 2020 – July 2020).

Response to reviewers: [PONE-D-21-39709] - [EMID:ef46cc445678c36e]

Dear Dr. Junhu Dai,

Thank you for allowing us the opportunity to revise our manuscript. The comments from the two reviewers were extremely helpful and made the manuscript much stronger. Based on their suggestions, we incorporated most suggestions to our manuscript.

Below, you will find a letter detailing how we dealt with each comment of each reviewer. Where applicable, we state what we have changed and the location in which you will find our modifications in our updated manuscript.

We address each reviewer comment (**C#)** in individual answers (**A#**) to keep everything organized.

Reviewer #1:
C1: The authors made an interesting work, continuously filming the surface behavior of crayfish which were thought before to stay mostly or totally in underground burrows. They registered activity both during the day and night along with daily environmental factor variations and analyzed their behavior through the video recordings. While most behavioral studies in crayfish have been done in laboratory settings, this one contributes by rescuing ecological context and opening new perspectives of research. It is mostly well done but there are three general issues to be considered:

A1: Thank you for the generous comments. We appreciate your suggestions and we have attempted to address all your comments below.

C2: One of the big contributions of this work is to enable discrimination of individual behavior, which is not a simple task in natural observations. Individual data presentations are fine but I suspect that when average analysis are presented, some or most of them are not considering that one individual (#2) with more than double the number of data collection is biasing the results. This can be deduced from some graphs while others do not let us know because description of average calculations are missing in Materials and Methods.

A2: We agree that analyzing behavioral data presents several issues and that this issue can be exacerbated by having an unequal sample size (263 hrs of footage for crayfish 2 vs 40 hours for crayfish 1). This is why we have chosen to present the majority of our results in terms of the percentages and not raw values. Reporting this information in terms of the raw values would certainly bias our results, as you suggest. However, by reporting this information (Figures 5, 6, and 7) in percentages, we believe that this is the most appropriate way to report our results and adjust for the bias. Furthermore, our manuscript reports relatively few true statistical analyses (and instead opts for more descriptive statistics of what we observed). This aligns with the primary goals of our manuscript—to describe the behavioral diversity of this elusive crayfishes and demonstrate how this new methodology can be lucrative in the field of crustacean behavioral ecology.

C3: While behavioral analysis can be made when these crayfish are on surface, nothing can be stated of their underground times. In this sense, it is an error to assign "inactivity" for underground times.

A3: We agree. Our manuscript can only report of the surface activity, and not any potential underground behaviors. We have altered our language throughout the entire manuscript to account for this comment. We have changed any discussion of "inactivity" to "inactivity at the surface".

C4: Discussion needs to be improved.

A4: We have taken several of your comments regarding topics that require additional discussion and expanded on them.

C5: Study Site: More basic description is required of the study area around the filming spot, to better explore the results. It is said that most species (tertiary and secondary burrowing crayfish) inhabit lotic and lentic environments but it is not clear how far are the Primary burrowing species of this study from water bodies, for instance. It is said in the Introduction that "Lacunicambarus thomai is a burrowing crayfish species with a high propensity to inhabit burrows in marshes, roadside ditches, and flooded fields (38). Populations of L. thomai often live in localized colonies with conspecifics and inhabit burrows that are relatively simple but can nonetheless be up to 1-1.5 m deep (38,39)". However, nothing, no information is provided of the specific area where the study took place: proximity from rivers, vegetation types and cover densities, average ranges of daily environmental factor variations, Latitudinal coordinates.

A5: We agree that information on our study site was lacking in the original version of the manuscript. We have now included a detailed description of the location in which we conducted this study (see Lines 127-134). Because our sampling location is on a residential property, we choose to not report exact coordinates.

C6: Burrows were selected and filmed. How far is one burrow from the other? Inform the average land area inside which the 6 burrows were located. One individual is associated to each burrow – is that an assumption or are there evidences that they are solitary? Furthermore, it is said that burrows may have more than one entrance (Line 67), how was this issue treated here?

A6: We have updated this information within our manuscript. We now provide information regarding how far away one burrow was from one another (Lines 131-132), as well as our assumption that each burrow was occupied by a single crayfish (Lines 141-144). We also clarify how we dealt with the issue with burrows with more than a single entrance (Lines 151-153).

C7: It is said in Statistical Analysis that none of the independent variables (humidity, temperature, and time) were strongly correlated (all $r < 0.6$). A representative graph depicting an

average 24h variation of environmental factors in the studied season would be informative in Supplemental Material. How was time included in the models, as a categorical or continuous variable? A continuous variable with linear increase (such as a sequence from 0 to 23) would create artificial associations in the model. Please evaluate, based on the statistical parameters found in Table 3, if it is not enough to use a simple model without interaction between time, humidity and temperature to understand the influence of environmental factors on the surface activity. The reason for this question is that the complexity of the best-fit model seems to have inhibited any discussion about the analysis in the end.

A7: We have now included 4 new figures in the supplemental materials (Figures S1, S2, S3, and S4) which display the relevant environmental data during our study period.

In the original manuscript, we reported that there were no strong statistical correlations between humidity, temperature, and time (as you mentioned). In our environmental analysis, time was coded as a continuous variable. We tested for the correlations beforehand to potentially deal with any artificial associations that you hint at. However, because of the low correlation values, we decided to proceed with using time as a continuous variable. Furthermore, the fact that time is not a strong predictor of activity (Table 4) suggests that there is no underlying association between these variables.

Our best fit model was quite complex, as you note. It was a model with 3 single parameters, and two interaction parameters. This why is we not only conducted a model-selection procedure (results in Table 3), but also the multi-model average procedure (results in Table 4). The results from our full model averaging technique allow us to evaluate all of the models together and construct effect sizes and weights for each of the terms in our model. This is why we come to the conclusion that humidity had the strongest overall effect (Table 4) compared to the other models. We have now included a better discussion of this result in our discussion section (see **A14**).

C8: The Results section start with “Hourly Activity” (which should be “Hourly surface activity”) and Figure 4, but there is no explanation as to how this was calculated. It is explained in Statistical Analysis that for an individual to be considered active on surface in one specific hour and day, it needs to be seen in any time point within that hour in that day. Then, how was the group average/percentage calculated taking into account that each contributing individual was registered for a different number of days? Individuals that had more filmed days should not weight more. Finally, Legend Figure 4 needs to inform that averages were calculated taking into account all individuals and all days.

A8: We have changed this section to “Hourly Surface Activity” as you suggest.

We have clarified how this was calculated in the methods sections (Lines 201-209). Again, we report these values in terms of percentages, and not the raw number of hours each crayfish was active to avoid any potential sampling biases as you suggest.

We have also updated the legend for Figure 4 to include those averages were calculated by taking into account for all individuals and all days throughout our study.

C9: An explicit description of calculation should also be added to "percentage of time spent active throughout daytime" and " proportion of time spent on surface". In Table 5, how was the "mean duration" of each behavior calculated, taking into account individuals and number of days each individual was filmed?

A9: We have updated our methods to also include information as to how we calculated these percentages. By reporting these values in percentages, we avoid any issues based on the number of hours each crayfish was sampled and filmed.

We have updated the legend in figure 5 to describe that our mean durations were calculated based on all crayfish. This data is reported to be entirely descriptive in nature, and therefore we believe that reporting such means are the most informative way to describe these different behaviors.

C10: In Figures 5, 6 and 7, it is interesting to show the "combined" proportions. However, it is biased by individual 2, which was filmed for more days. Could an unbiased calculation be made here?

A10: Reporting our results in terms of percentages is the best way to provide an unbiased calculation for these figures, which are primarily meant to be descriptive and exploratory in nature. If we would have reported the data from the raw amount of time that each behavior/activity was performed, then this would certainly be biased. This is why we chose to reported the data in terms of percentages throughout the manuscript.

C11: In Figure 8 the number of observations is again biased to individual 2.

A11: Yes, Figure 8 (but not 5, 6, and 7) is biased based on the increased number of observations from individual 2. This figure (and the analysis in general) are describing trends in whether or not a specific behavior was more or less likely to occur at day versus night. We have included information regarding this issue in our manuscript (Lines 201-203). Anecdotally, if you look at the percentages of all behaviors reported in Fig 5, 6, and 7, there is a similar degree of surface activity/behaviors being exhibited by each crayfish, which implies that this bias may be minimally impacting our results.

C12: Figure 5 and associated text: comparison should be between "surface" and "underground", not between "active" and inactive" because nothing is known about what the crayfish are doing underground. The same for Figure 6: "nighttime on surface" and "daytime on surface". In Line 281, replace " active" by "on surface" in "The percentage of time that crayfish were active

during the daytime”. In Line 283, “Regarding nighttime activity on surface”. Legend Figure 6: “percentage of time that each individual crayfish spent on surface during the day and the night”.

A12: You are correct; we do not want to mislead the readers. Now, all of our figures have been accounted for the fact that we are only reporting surface activity and that we cannot comment on underground. Activity. Figures 5 and 6 are now updated accordingly. We have also changed the legend for Figure 6

C13: Discussion: In contrast to Bearden et al. (2021), this study brings more information about the behavioral complexity of this particular crayfish species. However, the lengthy discussion is mainly descriptive of results. The authors should explore, for instance, what was found in statistical analysis and how could this be connected to the specificity of the studied environment, to take full advantage of the in situ study. Another suggestion is to take Bearden et al. (2021) as a reference, discuss how the results are constrained by the particular season and microhabitat that was covered in this study.

A13: We agree that we could have expanded on our discussion section. Based on many of your suggestions below we believe that the discussion has significantly improved.

C14: Humidity was indicated as the most important factor modulating surface appearance in crayfish. This variable, as well as all others were collected from a meteorological station. Any thoughts about the validity of using only macro-environmental measurements in association with behaviors that are restricted to the spatial scale of the entrance of a burrow?

A14: We agree that the macro environmental variables that we relate to surface activity are far from ideal for such studies. We have updated our discussion to point this out and suggest that future studies take into account the variables potential micro-environmental influences within and around the burrow entrances (Lines 406-422).

C15: Temperature was shown to be a strong predictor of surface activity. A strong suggestion for future studies is to also consider underground temperature in this analysis. For instance, it has been shown in endothermic subterranean rodents that a combination of external and underground temperatures predict better the episodes of surface emergences (Jannetti et al., Conservation Physiology 2019 v. 7, p. coz044). It is reasonable to assume that similar influence is potentially valid for these crayfish.

A15: This is a great suggestion to add to our discussion section. We have now included that looking at the underground temperature variation is a fruitful area for future directions (Lines 416-422).

C16: This crayfish display clear postural signatures that enable behavioral identification through

the relative position of body coordinates (Figure 3). This could potentially be used in future automated video analysis of activity patterns.

A16: That is a great suggestion to add the discussion. We have included these postural changes to be used in the identification of automated video analysis in the future (Lines 511-513).

C17: Fig.2C: view of the camera

A17: We have edited this sentence accordingly.

C18: Line 248: remove extra "were"

A18: We have removed the extra "were"

C19: Line 319: "every single behavior was more likely to occur during day or night compared to another" An alternative could be "daily phase"

A19: We believe that the current wording provides for clarity because "daily phase" may be confused with "day".

C20: Table 4: what does this mean? "the activity of L. thomai was negatively related to the activity of
crayfish."

A20: This was a typo and should have read "Thus, the activity of *L. thomai* was negatively related to the degree of environmental humidity." We have made this change to the Table 4 text. Thank you.

Reviewer #2:
C21: I understand that behaviour of strictly burrowing crayfish is difficult to study, so the information presented by the authors is surely interesting, and novel. However, the entire manuscript is based on only six individuals that were not even characterized. So, data are very preliminary and should be interpreted more cautiously. Behavioural categories should be also analysed together, considering that are dependent data. I am sorry not to be more positive, but a more specialised journal seems to be more appropriate for the manuscript.

A21: We agree that the fact that our study being only conducted on six individuals is a limitation to our study. Low sample sizes are typical for this type of work, because of the time intensive nature and complexities of naturalistic studies (see 6 individuals reported in Janettii et al. 2019) Although this work was only conducted on six individuals (that have unknown demographic information), we believe that the amount and nature of our data is worthy of publication.

In the previous and updated versions of our manuscript, we attempt not to overstate our results as they are preliminary and exploratory in nature. Furthermore, although we do not report the demographic data from these crayfishes (sex, etc), there is no data that has alluded to sex differences in burrowing crayfish behavior. We do agree that this is an interesting and important angle for future studies though, so we have included that this information should be investigated in the future (Lines 507-508).

Despite these limitations, we still believe that we provide a novel methodology and interesting results (which reviewer 1 highlights) that will be of interest to a wide audience. Because the scope of PLOS ONE is to publish papers based on their scientific validity and methodology, we believe that our study is of interest to an audience outside of smaller, taxonomic focused journals.

We respond to the comment on behavioral categories being analyzed together below (see **A27**).

C22: Lines 50-52, 109: for the readers it would be interesting to specify that these species are North American

A22: We have edited Lines 50-52 to clarify that these are North American species (Line 50-51)

C23: Line 67: maybe opening is more suitable than portal

A23: Portal is a term burrowed from the literature on mammalian burrowing behavior and is widely used to refer to crayfish burrow openings. Therefore, we prefer to keep this language consistent with prior published papers. We have included a few examples below.

Glon, M. G., Adams, S. B., Loughman, Z. J., Myers, G. A., Taylor, C. A., & Schuster, G. A. (2020). Two new species of burrowing crayfish in the genus Lacunicambarus (Decapoda: Cambaridae) from Alabama and Mississippi. *Zootaxa*, *4802*(3), 401-439.

Loughman, Z. J. (2010). Ecology of Cambarus dubius (upland burrowing crayfish) in north-central West Virginia. *Southeastern Naturalist*, *9*(sp3), 217-230.

C24: Lined 128-130: so how many burrows were checked before selecting only six? How about the density of the burrows in the study area?

A24: We have now included much more information regarding our study location. Based on the relatively small number of burrows at the study location, we choose to only focus on a small sample size but to collect as much data as possible on each of these adult individuals.

C25: Line 132: crayfish could have been attracted out of the burrows with some baits after the

footage to characterize them (for hunt behaviour it is reported they leave the burrow for example)

A25: Yes, that is true. Unfortunately, we did not capture these individuals. We have included information how future studies need to explore how different demographics may exhibit such behaviors differently (lines 50-51).

C26: Line 182: were the behavioural data checked for normality?

A26: Yes, all data and model fits were checked for normality. We have now included this information in the manuscript (Lines 197-199).

C27: Lines 218-222: those behaviours are dependent each other, so it is better to analyse them together because when crayfish are guarding, for example, they are not feeding or hunting

A27: This comment highlights the complexities of working with behavioral data, because an organism cannot perform more than a single behavior at once. Therefore, this is why we chose to primarily focus on broad, descriptive statistics throughout our study. Because of the novelty of these findings and the potential impact on our understanding of crustacean behavioral ecology, we believe that this is the proper way to analyze report our data at this stage.

C28: Line 235: please delete during the study

A28: We have deleted this phrase.

C29: Line 270: please 5 not in italics

A29: We have unitalicized this number.

C30: I suggest merging Fig 5 and 6 in one figure

A30: We prefer to keep these figures separate as to avoid confusion between the messages of figure 5 (observed at surface vs. underground) and figure 6 (observed at surface during the day versus observed at surface during the night).

C31: Table 5: please report all the duration in seconds. Moreover, please change during with duration in the caption

A31: We have changed the table to report each behavior in. We have changed during to duration as you suggested.

C32: Tables should be better drafted and presented

A32: Without any comments on the issues of the tables, we are unsure what to change base.

C33: Line 343: please correct five

A33: We change made this change.

C34: Line 345: please better rephrase this sentence

A34: We have rephrased this sentence accordingly (Lines 366-368).

C35: Line 356: Loughman et al. 2018 is not present in the bibliography; 46 is Loughman et al. 2015

A35: Thank you for noticing this issue. We meant Loughman et al. 2015 and we have made this change.

C36: Line 371, 448: please consider that only six individuals were observed, so I suggest being more cautious in this statement

A36: We agree that we need to be more cautious with these statements. We have added additional information to these sections based on your comments (see Lines 394-395 and Lines 494-495).

C37: Line 381: please do not use intricacies but richness or diversity

A37: We have changed the work intricacies to richness as you have suggested.

C38: Line 415: please correct "is required"

A38: We have changed "are required" to "is required"

C39: Line 436: I think it is "use its claws to push the mud"

A39: Thank you for catching this error. We have fixed the typo.
