# Supplementary material for: On the surface or down below: Field observations reveal a high degree of surface activity in a burrowing crayfish, the Little Brown Mudbug (Lacunicambarus thomai)
Source: PLoS One. 2022 Oct 14;17(10):e0273540. doi: 10.1371/journal.pone.0273540 (PMC9565396; doi:10.1371/journal.pone.0273540)
Supplement: Attachment — Submitted filename: response_to_reviewers_2.docx [file pone.0273540.s014.docx]

Dear Dr. Louis-Felix Bersier,

Thank you for allowing us the opportunity to revise our manuscript. Your comments were extremely helpful and made the manuscript much stronger. Based on their suggestions, we incorporated most suggestions to our manuscript. Below, you will find a letter detailing how we dealt with each comment you provided.

We address each comment (C#) in individual answers (A#) to keep everything organized.

**Editor Comments:**

**C1:** This level of precision is not necessary. I recommend rounding at unity here (21% to 69%)

**A1:** We have rounded these percentages.

______________________________________________________________________________

**C2:** Could be abridged as "tertiary-, secondary-, and primary burrowing species"

**A2:**  This is a good suggestion, we have changed the text accordingly.

______________________________________________________________________________

**C3:** delete

**A3:** We have deleted the words “the setup of” here.

______________________________________________________________________________

**C4:** I guess "... unable to report ..."

**A4:** Yes, you are correct. We have edited the text here.

______________________________________________________________________________

**C5:** Meaning not clear to me. If it indicates that this behavior occurs mostly during the night, it should not be part of the description in the table, but should be stated in the results.

**A5:**  This statement (more exposed during the night) has been deleted because we agree that it is unclear. We meant to mean that when the crayfish exhibit the guard behavior during the night, they are typically more exposed out of their burrow portal. But we did not quantify this, so we chose to delete it from the text.

______________________________________________________________________________

**C6:** The images are very useful and raise a question since these two behaviors are very similar. As a non-specialist, I would like some words (and possibly a reference) that explain and justify this distinction.

**A6:** After some discussion, we agree with all of your comments regarding our denotation of these behaviors of “guard” and “rest” behaviors. We now refer to both of these behaviors as “rest” and separate them based on “rest-claws open/rest - open” and “rest-claws joined/rest – joined”. Although rest – closed may represent a guarding behavior, we believe that because this work is preliminary, we are keeping the language simple. The manuscript and figures have been changed accordingly. We believe that this language will properly describes the behaviors of the crayfishes would making any presumptions on the function of the posture.

______________________________________________________________________________

**C1:** There may be an unimodal relationships with these variable, reflecting optima. Did you check for this possibility ?

**A1:** We did check for this, but because there was not as much variation in the environment variables, we did not find any optimum relationship between activity and such environmental variables.

______________________________________________________________________________

**C1:** Personally, I would place the Tables 3 and 4 in the Supporting Information (only a suggestion).

**A1:** We have moved Table 3 and Table 4 to the SI as you have suggested.

______________________________________________________________________________

**C1:** This should be part of the Method section, e.g., on line 216, in the parenthesis "(

**A1:** We have moved this section the methods section as you suggested.

______________________________________________________________________________

**C1:** Same remark as for the Abstract.

**A1:** All of our percentage values have been adjusted to be reported in the way that you suggest.

______________________________________________________________________________

**C1:** I do not see the correspondance between the percentages in the text and in Fig. 5. Please check if it is correct or provide better explanations (are the colors reversed in the figure ?)

**A1:** Thank for noticing this mistake. Yes, the colors on the figure legend should be reversed. in Figure 5. We have fixed this accordingly. Now the numbers and percentages add up correctly.

______________________________________________________________________________

**C1:** Again, I have problem with the values in the text and in Table 5. Crayfish 4 is the only one that foraged during the day. From Table 5, this happened only once, with a total of 360s, which is clearly not equal to 0.34 hr. Please explain.

**A1:** After checking back at our data, you are correct, we mistakenly calculated a larger percentage for the daytime activity of Crayfish 4. This change is now reflected in the manuscript and in the Figure 7, and Table 5. We re-checked the other crayfish as well and the results are correct for them.

______________________________________________________________________________

**C1:** Same remark as for Tables 3 and 4. The results are different but redundant with Fig. 7.

**A1:** We have moved this Table (Table 5) to the SI as you have suggested.

______________________________________________________________________________

**C1**: But Fig. 7 shows the opposite pattern! Table 5 support this.

**A1:** Thank you for catching this error. This was a typo and now correctly states that “The majority of their time was spent in the relax position during the night, and relaxing was more likely to occur during the night compared to during the day.”

______________________________________________________________________________

**C1**: I think that "proportion" is more adequate here.

**A1:** We have reworded this to proportion based on your suggestion.

______________________________________________________________________________

**C1:** Again, I would place this Table in the SI, as the main information can be visualized in Fig. 8.

**A1:** We have moved this table to the SI based on your suggestions.

______________________________________________________________________________

**C1:** This relates to my question concerning Fig. 3 (line 183). Does reference 51 separates these two behaviors based on claws' position?

**A1:** See A6.

______________________________________________________________________________

**C1:** At this point, I am uncomfortable with your choice of words (relax and guard) for the two behaviors based on position of claws. Perhaps "guard - claws open" and "guard - claws joined" may be more appropriate?

**A1:** See A6.

______________________________________________________________________________
